# Supplementary material for: Patients’ perceived quality of care and their satisfaction with care given for MDR-TB at referral hospitals in Ethiopia
Source: PLoS One. 2023 Feb 2;18(2):e0270439. doi: 10.1371/journal.pone.0270439 (PMC9894439; doi:10.1371/journal.pone.0270439)
Supplement: S1 File — (DOCX) [file pone.0270439.s001.docx]

# VERBATIM TRANSCRIPT OF THE INDEPTH INERVIEW WITH MDR-TB PATIENTS

| Interviewee type | The principal investigator moderated all the audiotaped discussions with in-depth interview participants. Questions are asked by moderator (**Mod**) & answers by the key informant (**KI**). |
| --- | --- |
| Respondent-1,  Sex-Female | **Mod**: Thanks for volunteering to be part of this study. How did you first understand that you have MDR-TB?  **KI**: Do you mean first when the disease started?  **Mod**: Yes, were you ever treated for TB so far?  **KI**: Yes, the six month treatment.  **Mod**: Was that unsuccessful.  **KI**: Yes. I received the six month treatment, and it became unsuccessful. I was ill just at the end of the six month treatment. I was very ill. Then I told the doctor that I was ill. When I told him that I was coughing, he told me that he sends my sputum for me. Then he sent my sputum to Nazareth. Then exactly after seven days the sputum returned back [respondent wanted to men the sputum result returned back]. TB he told me, TH that is resistant to drugs. And he told me that I go there [to the TIC at Nazareth] and start the treatment. so as he tol me I came here just on the same day that was Thursday. I came and started my drugs.  **Mod**: when you first started treatment at hospital and before you were linked to health center that is nearby to your home area, how do you describe the discussions and communication you had with your provider on the type of drugs you take, on the places where you can continue drugs and other issues and concerns you had? Means were you part of the decision making process?  **KI**: It was not decision but they tell us that, when you are sick now you started this medication, when you are sick you discuss with the health center and you return back to this center. When they could not support you come here and all available things we treat you they said. Yes when we get sick, sick we come here and it is here that we are treated. Until the end, now I am approaching to complete the treatment.  **Mod**: How do you describe the social and economic impact that caused unto you because of your becoming MDR-TB patients?  **KI**: very much it separated me from people. It means living alone, sitting alone, there is no work that you cannot work, no work is done, it is just lonely living!  **Mod:** What is the main reason for your living alone? Is it because people discriminate you or you just take care of others?  **KI:** It is just for the disease, for the disease as it is transmittable I take care of others.  **Mod:** How do people perceive about you when they know that you have MDR-TB?  **KI:** Yea, at our disposal people do not know much. They do not know that the disease is difficult. But as I now the disease I refrain from them. They do not see it as serious disease.  **Mod:** You mean that the public does not know the disease?  **KI:** They do not know. Especially this disease they do not know at all. What kind disease are suffering from that people that ever catches tuberculosis is treated and cured soon they ask me. Your TB you are not getting cured at all they say to me. For the whole two years why you take the drugs they say. This is not TB they say to me. Therefore my problem, I have been suffering, I have seen, I know the problem but you should not be sick like me I tell them.  **Mod:** Do you have children?  **KI:** No I do not have children. I live alone.  **MOD:** At this hospital there are transports and nutrition supports to enable patients continue treatment. How do you explain that support?  **KI:** We are given from the beginning up to the end. We come every month and we are given transport cost, we are given 120 birr (equivalent to 5.5 USD), and other benefits we are given when we come monthly.  **Mod:** How do you describe its adequacy?  **KI:** Just we take what we are given!  **Mod**: I know that you take what you are given. But is what you are given enough from your perspective as a patient?  **KI**: I say that it is enough for me.  **Mod**: How do you describe your satisfaction with the clinical care you are given both at this hospital and at the health center?  **KI**: Here it is just as you see it. I returned back immediately after transferred to health center. After this as there is no drugs I take the drug I used to take.  **Mod**: I think the reasons for you to be readmitted this time is due to the drug-related side effect than the TB disease.  **KI**: Yes.  **Mod**: How do you describe the cleanness of the compound also?  **KI**: The compound is clean.  **Mod**: How about the clinical care services you are given?  **KI**: the clinical care, they give us drugs but they are not available on demand. They are not available when we become faint. If they are to come, they come only after calling them.  **Mod**: What is the most difficult thing that you encountered in the course of your treatment for MDR-TB?  **KI:** My experience is that there was incidence where I was seriously ill and brought by car. At that time there was no Doctor. Then I become faint and was near death. After I lost my consciousness, he came after five hours ad after he was called. At that time it was mean that I was dead. At that time if they were here and if you are treated on time telling him the pains you have I believe that you can get relief. But as they do not come on time/on demand/ when you get sick at day time or at night time they come indeed only after calling to them.  **Mod:** is there anything that you want to rasise that I have not raised?  **KI:** No anything that I add. They come in the morning and give us injection that is it.  **Mod:** Thanks for your time. |
| Respondent-2  [young male patient] | **Mod**: Thanks for volunteering to be part of this study. How did you first understand that you have MDR-TB? Did you take anti-TB drugs before?  **KI**: First yes, for six months. I completed the six month treatment. But the cough decreased but did not disappear completely. There was also expectoration and cough despite the treatment. Finally I was asked to submit sputum and when I submit the sputum, I think that she might have sent it to this hospital and it was said that it is just as it was before. Then she told me that I had to start the eight month treatment regimen. After I took the drugs for fifteen days she aske me to submit sputum and I think that she sent it to here and then it was said that it is tuberculosis resistant to drugs. Then she sent me to this hospital.  **Mod**: Now you are young patient. What are the social and economic problems have you encountered because of being MDR-TB patient?  **KI**: Yes before I used to cater by running here and there. But after I was diseased there is problem, people do not have good perception and also I do not have the strength to work as I used to be.  Mod: You mean that being MDR-TB patient has impact on social and economic issues?  **KI**: Yes  **Mod**: There are supports made to enable patients continue treatment like food and transport. How do you describe the transportation and nutrition support that you are getting from the hospital?  KI: That is you can do nothing and you say that it is enough. What can you do after all.  Mod: I know that you take what you are given. But my question is how do you describe what you are given like its adequacy and quality?  KI: It is good.  Mod: Were you admitted to this hospital at the start  KI; Yes I was admitted.  Mod: Was the food you were given enough.  KI: Yes, it was not enough.  Mod: What about the food you are given for a month, it is enough.  KI: for the whole month? No it is not enough. Unless for patients who have supports from the side like by parents or relatives, the food given by hospital cannot be enough for one month.  Mod: How do you describe the clinical care you get from MDR-TB care providers like during times of emergencies?  KI: That is good.  Mod: What is the most difficult challenge you faced during your treatment for MDR-TB?  KI: It is said that treatment is given to patients but there patients not improving at all. There are also patients thay do not return home alive.  Mod: How do you describe patients improvement inn the course of the treatment?  KI: It is good but there are also incidences whereby patients get sick and return back to the hospital.  Mod: How do you describe the cleanness of this compound?  KI: it is good, they clean it daily.  Mod: Is there anything that you want to add that I have not addressed?  KI: yes, on the food it is not tasty at all. It is good if the food is prepared in the way that a patient can eat.  Mod: I understand that you have complaints on the food?  KI: yes. When we tell them, they say that they do not have enough supplies from the central level. Just not to stretch workers here, we simply keep quite.  Mod: Thank you for your time and the information. |
| Respondent-3 | **Mod**: Thanks for volunteering to be part of this interview. I have couple of questions regarding your experience as an MDR-TB patient. Are you part of the decision made about your treatment for the MDR-TB you have? Means that are your voices heard?  **KI**: I mean it is just three month since I started the treatment. the type of the services I get are bed accommodation, and the bed is clean. But on the food there is problem. I am not given the food that I need and good for for persons like me treated for MDR-TB. Every day we eat the same type of food every day and the same is true throughout the week. I am attending the drugs and the drugs have brought changes on me. Moreover I am living with HIV virus and am taking drugs for that. Since I started drugs for both diseases, you understand that I need additional foods. The food I am given is not adequate and I feel hungry at night. It looks like this and the physicians also follow me closely and takes good care of me. Till now it looks like that.  Mod: You have told me that you are an MDR-TB patient and also that you told me to have another additional health problem. How does these two diseases impacted your social and economic life?  KI: Before I was formally employed. I am soil laboratory technician. I have degree in that, from the total income I used to get, as I was employed to a private company, my income was discontinued since I caught this disease. I could not also support my family, so that it brought high problem on my life. It is that!  Mod: Do you have relatives/ household members who are dependent on you?  KI: Yes there are family member who are dependent on me. They are my mother and my father, as I work at private company everything is discontinued. I have one sister, she also has family but she saves some money and gives it to them. It means that we are on problem until I get out of this with health and return back to work.  *[the patient looks very hopeless and I was to sooth him through counseling,…the situation is very sad and it was very touchy, I discussed with the patient MDR-TB can be cured even in the presence of HIV/AIDS and you will cure of the MDR-TB and return to your normal work*  ***KI****:* The treatment is on me as I am getting strong there is no much problem but on other patients as the health care providers are not available here the whole day, they do not attend the patients and they come only after the patient enters into come. That situation has been endangering the patients. I have been seeing many cases and there are many patients who lost their life due to absence of care. For example at the evening, night and afternoon when a weak patient comes, the physicians here come through telephone call. Do you understand there is no one who asks you whether you are improving and for presence of some problem. The food is also as I told, we eat same type of food throughout the week. The problem with food is not the way it is prepared but they say that they do not have the supplies. Only the sanitation is good  Mod: Thank you for the information, I there another issue that you thought would have been raised?  KI: The things I say as good here are for example there are protective devises used ( patient points to face masks), sometimes when relatives come to salute you there are times when the relative returns back without greeting you because here they say that there is no supplies for that and also the issue of the drugs especially the TB drugs it is very difficult. For example I with my kilogramme first when I came I was forty kilogrammes and now I am around 45 KG. From the entire drugs one drug is very difficult; at least until three hours (means after taking the drugs) it puts you into something different life. It causes sense of vomiting and disturbs your mind. So this and this things It is not a drug given for someone who is weak patient and needs modification. But sometimes the physicians from many angles they are good. But sometimes when there is no one and the patient is hurt there is a scenario whereby they come only after telephone call. Otherwise the cleanness is good. But the clothes that there is only one blanket and one bed sheet, the situation is very cold and one bed sheet you put on the sponge and the blanket you wear and this things need to improve.  **Mod**: Thanks for the information  KI: Ok thanks |
| Respondent-4,  Sex-Male | **Mod**: Now you are taking treatment for drug-resistant TB. For every concerns you have do you get the opportunity to discuss with your providers regarding the decisions you need?  **KI**: First when I started taking anti-TB drugs, first I took six months treatment regime. I understand only after six months that I had MDR-TB. After six months I went to Asella hospital and I new that I had MDR-TB and then came to this Geda hospital. First I attended at private hospital when I took the six month’s treatment. When I ask the doctor about the disease and when I discuss with him when I say this this drugs he did not make good follow ups for me. Because of this I faced big problem and repeatedly came to this hospital. But thanks to God, first when I came I was very weak and at that time there was Dr Beyan some advise and then I get improved and rose up. From five patients that were admitted together to this hospital, only two of us discharged alive and three patients died of the disease. Three patient died. Also there are many patients that were died but for me may God be blessed that now I am attending treatment on my one year and second months’ time. Now for the follow up treatment I am attending my treatment at Asella. I can say that the sister giving me the drugs as my mother. For her I have the same respect that I have for my mother. She also advises me as I was about to commit suicide.  Mod: You are young and every person wants that you get cured from the disease, you are community asset.  KI: I do not hide it from you that because of lack of the knowledge, my sister caught the disease from me and she is getting the treatment at this time.  Mod: were you living in the same room at that time?  KI: No at that time she was living at Addis Ababa. She came to salute me and we spent together only three days. And as the problem is airborne she caught the disease.  Mod: was she sleeping in the same room as you at that time.  KI: Yes for the three days she was in same room with me. At that time I did not start this drugs and I was taking the six months’ treatment. I did not start the treatment for MDR-TB.  Mod: How do you describe the social and economic impact that the disease brought upon you?  KI: It posed many influences and it includes quarreling with my family. I was set alone in the rural area and I was seen as hated person. Even when I try to explain it for them no one accepts it and they say that there is no such disease. I was also labelled badly. I was living in a separate kiosk and also stabilized my mind and thanks God that I am alive today.  Mod: How do you describe the food and transport cost you are given by the hospital?  KI: What about the food? With respect to food we are very hurt! The disease decreases appetite. For three months I was eating only fried peas and check pea (shimbra). Otherwise I was not able to eat foods like the meat and vegetables. Because of the gastritis I had I could not eat food and also make me vomiting. No harm that was not inflicted by the disease on me. It disturbs my mind. I vomit and also vomit blood like that. I was not able to move as I was not eating foods. But thanks God that related to food after I came here first on January 8 2016 and I returned back as my paper seal was not right. I started treatment on January 10 and after that I could eat food little by little and after that after taking the drugs for one month the case whereby I was not able to eat half of what I should do, I started to eat half of what I should and I took the drugs for four months and then get tired once more. At that time different medications were made for me and after five months thanks to God that I am eating my food. Having passed through many challenges I about this disease let alone my family but the whole society if there is someone who takes the message from me I am very interested to teach others and to share the experience I passed so far. But the attendance and care given here at this hospital is very different from the care given at the treatment initiating centers and the care at health center is very weak. There are times when providers close their doors against us and abandon us. They do this being educated professionals. There are times when they deny us treatment for two and three days and we phone to this hospital and complain the situation and talk to sister and Bekele to talk the fact of God. So is the public learns and knows the problem the public will not be hurt by the disease. It is very good if it is taught through media.  Mod: Is there anything that did not raise that you want to raise?  KI: There is no much thanks that I can add,…there money I spent on this disease can is too much wich can be used for many things . an educated person knows much things and it is very important to educate the public. When I refrain from them they say to me as ,…he refrains from us to die they say. The urban knows something but the rural community knows nothing about the disease. It is very important if the public is taught on this problem and also I thank you for making such questions to know the problem.  Mod: Thanks for participating. |
| Respondent-5 | Mod: Thanks for volunteering to be part of this study. How do you describe the socio-economic impact that the MDR-TB posed on you?  KI: It has beenvery difficult for me, I have not ever been diseased before this disease.  Mod: Is this case of TB the first course of TB you diagnosed with?  KI: Yes, there is also economic problem and I used to work on daily labor work with privates. It may be due to that case because of the problem I have. I eat when I get and do not eat when I do not have something to eat. Because of this the disease caught me and when it caught me it immediately collabsed me (tset aregegne). First I became only bony and I couldnot even talk and my eyes became yellow and also the whole my body became yellow. Then I went to Asella Hospital and they said that it is TB. And they gave me dugs for two months, but as there were not any improvement they asked me submit sputum and the sputum result they said that I was not improving and even the case has been more severe and told me to go from Asella to Adama. My brother was working with them [Asella hospital] and they allowed us to use ambulance. when the ambulance brought me here, I came to Adama. But they ordered me to go private and get x-rays and blood test. But as I could not walk my brother could not carry me alone and others helped him an they carried me to the private clinic and the x-ray and the blood was examined and then I came to this hospital [Adama TIC]. Then I was admitted and treated and then I got improved and when I get improved they ordered me to go back to Asella. I said okay and went back to Asella. When I went to Asella it is icy cold and I was going out every day for morning injection and then y. every day I came back from injection I was sick and sick every day. As such I again became very sick. Again as it was at the start my legs and hands became stiff and I could not eat food and I started vomiting. Then I rose up, telephoned them and came back here again.  Mod: How do you describe the nutrition support you get here?  KI: Food goes with the wealth you have. If you are rich you eat good f  Mod; I mean the food that you get from the hospital here?  KI: here it is a serious problem. ‘Shiro’ is not prepared with due care and some food like the vegetables we buy by our own cash and are preparing and eating. The ‘shiro’ has no any test of food, let alone a diseased person but the healthy person cannot test it. When they serve meat sauce it is just watery and there you may see a sign of meat but it is water. We could not eat that. All things do not have test, do not have ethics. There is serious problem with respect to food.  Mod: Is there anything that you want to add?  KI: there is also problem of clinical care here. While we are feeling pain we do not get doctor. We phone and make them called. Either they come or they do not come. To tell you the truth, here in this compound she is only one sister who prudently accomplishes here duty. She is good. When on duty she passes the night in this compound when it is her turn. But he [another male MDR-TB nurse], we do not get him. We pass day long with our pain and even if we die we die alone. The toilet is not emptied timely and it fills and spills over. There is many problems.  Mod: Much thanks for the important information and the infoemation is important to learn the situation that will benefit current patients and also those who will be diagnosed in the future. Much thanks.  KI: Okay. |
| Respondent-6,  Sex-Female | Mod: Thanks for volunteering to be part of this study. During the initiation of your treatment, did you discuss on issues of treatment, places where treatment is available? Was your voices heard by your care providers?  KI: When I became ill and came here, all the available things , about the disease, the treatment, how I should I attend the treatment, how I should get the treatment, the drugs and the injections and about the months all tings they have told us. They have advised us, at home also those living with us the disease is communicable and that you have to take care of others as you do for yourself that all they told us. the equipment used for drinking, with some utilities to prevent the disease from transmitting through breathing. They said that we need to have separate equipment and take care of others in the house. They told me not to interrupt the drugs and the injections also. They told me these all at the beginning and after I came here I started my injections. I took the injections here for about weeks and I took the injections properly and then they sent me back when my date was due.  Mod: Imagine that you are a young MDR-TB patient. How do you describe the social and economic impacts that the MDR-TB disease brought on you, if any?  KI: It hurt me very much.  Mod: were you discriminated?  KI: No such problem. I caught the disease while I was taking care of my husband. I haven’t ever got TB before. I did not also take any treatment that is called the six month treatment.  Mod: Who was the relative whom you were taking care of and that you caught the MDR-TB?  KI: That he was my husband. While I was taking care of him, I did not know the disease as both I and he did not have this disease before. They put him on the six month treatment and there they did not give us any precaution. No any advice that we were given for him also when he started treatment and I did not think that the disease transmits and in fact he is my husband and I could not abandon him because he is sick and I could not go away. But if were told that it transmits I could have taken care and he also would have taken care of me.  Mod: Is he taking the treatment now?  KI: Yes, he is attending the treatment. Even he has elapsed one year and two months by now. There is one month gap between me and my husband. At that time it made me a little cough. It was when he completed the fifth month of the six month treatment and when there was no improvement for him and when we did not have any option, we took him to another clinic and made him seen by another doctor. First the disease was hidden but finally it was found at the hospital. Then the hospital found the disease and then we were sent to this hospital and when we came here we were told that it is drug resistant tuberculosis. Then they told him that the disease was from another person with MDR-TB and they told us that the disease is communicable from person to person. Then he told them saying that ‘my wife has been living with me’ and he registered me here for follow ups and then I was called to the health center for follow ups. My husband demanded that I should get examined for the disease as I was taking care of him. They advised me to submit sputum and I said okay and it was when my husband was discharged from this hospital and followed his treatment at the health center at Asella. The hospital examined my sputum and they said that a small thing is seen but it is not seen very well and they said that the type of TB is similar to my husband’s TB and they told me to go to Adama and I came here and started the treatment. here also I was examined and they said that everything was normal and I decided that health is a priority and accepted their advice and started the treatment. I decided to start the treatment early and get rid of it and said to them okay. Then I started the treatment and I am taking up to this day.  Mod: how do you describe the nutrition support that is given for MDR-TB patients like you?  KI: That thing we thank the government and also that may our country become peaceful country, we also owe our government thanks because currently there are many problems that we have at our home. There are many who live in rental house and thinking that all the government has for patients like us who is sick and cannot work to earn the government has been supporting us with something like money and others.  Mod: is that support sufficient?  KI: That whatever it is we are given it we say sufficient. But there are patients more poor than me and for whom the food id not enough for the whole month. This is because there are some patients who do not have someone to assist them and they also cannot work to get money. They are in rental house and they pay house rent and as you know the house rent is paid my going here and there and work labor work to pay the house rent. But after catching this disease she cannot work the labor work also because she cannot have the strength and also the drugs are very difficult there is also injection and when she takes these drugs she have to have something good food to eat. What it means that it may not be sufficient. For me thanks to God that as it was not much severe on me and as we also have some at home I say that it is enough for me but for others I do not know the reflection of others. It may not be enough.  Mod: Thanks for participation and that the information you gave me is very important to improve the service fot MDR-TB patients like you. |
| Respondent-7 | Mod: Thank you for volunteering to be part of this study. What are the social and economic problems, if any, that you encounter as a result of becoming MDR-TB patient?  KI: One thing my being separated from people makes me feel discomfort.  Mod: Do you mean that people discriminate you?  KI: No it is I want them not to be like me and I take care for them. It is to safe them not to Cath the disease and from my children also not to expose them even they want to come closer to me but I go away from them they want to sleep with me at night and also when I go asleep they come and sleep at the side of me and I stand up from their place and sleep another place.  Mod: You do good thing because the disease transmits through air.  Mod: How do you describe the nutrition and transport support you get from the hospital? How do you feel as in the eyes of an MDR-TB patient taking the support?  KI: In the eyes of the patients it is good because we do not have money for transport and also we cannot mingle with people to work and get money. The transport id good but for those who cannot work and have something at the side, it is not enough. There are mothers taking care of her children who do not have father and also for family who do not have someone to assist them it is not enough. For someone who have someone to assist may be enough. For example if I take myself as an example I am separated from my husband and I am taking care of my two children and the food I get from here is not enough and alo I do not have the strength to go for work. I say and afraid that I may feel pain and also I say that the disease may relapse and I refrain from being exposed.  **Mod**: In conclusion you mean that it is not enough?  **KI**: Yes  Mod: How do you describe the clinical care you are getting from this hospital?  KI: They care it is good, they are giving us drugs and also they are giving us the injections, the care they give us is good.  Mod: As an MDR-TB patient what are challenges that you have come across that may be important for service providers and the programme?  KI: Above all the drugs are very miserable. Especially the last two drugs are very dangerous and they are not even fair to for mankind. Indeed it makes my blood vessels and my eye burn up. It is very difficult and I am happy if those drugs are changes to injections or other drugs. It is the drugs if I drink mild I vomit when I eat it poisons the food and I vomit. I feel as if my body is tied us with rope and my mind does not work and my eyes cannot see at that time.  Mod: Much thanks for the important information that you gave me and I advise yo to continue taking care of your children. Much thanks.  KI: Okay. |
| Respondent-8 | Mod: Thanks for volunteering to be part of the interview. By this time you have been on treatment for close two years. How do you describe the social and economic challenge that you encountered as a result fo becoming MDR-TB patient?  KI: No, on my work there is no any influence that was caused by the disease.  Mod: Were you engaged in your usual work?  KI: Do you mean out of my home?  Mod: Yes  KI: No, as do not have the strength I cannot work outside except the household works. But in my home it is me who cooks for the family.  Mod: Do you have dependents that are supported by you?  KI: Yes, I have one student child.  Mod: Who supports that child with the school, food and clothing?  KI: For him, he does some work after school and for my household expense he supports me to some extent. And also what we are given from here monthly they give us oil, lentils, milk powder and grain flour and also transport.  **Mod:** Is that food and transport support sufficient?  **KI:** No, it is not sufficient; I mean it is not sufficient. How can 10 kilogramme grain flour be sufficient, it is not sufficient.  Mod; How about the transport?  KI: transport first it was 200 birr and in the meantime it reduced to 180 birr and we written and submitted complaints and they said that they will correct it but it may be corrected in the future.  Mod: From where are you coming now?  KI: from Metahara.  Mod: And you say that the transport support is not enough.  KI: yes it is not enough. Even now ours become deteriorated, it needs receipt and also that if we cannot get receipt we should go to the new bus station and come from there and there is also transport cost from bus station to home.  Mod: How do you describe your satisfaction with the clinical care you are given here?  KI: For the clinical care they care for us very much. Even I there was…let I not speak that*...[the informant swept and she narrated how here young son was stabbed to death in a social conflict while she was away from home for MDR-TB treatment]…men killed my 29 year old son* I mean while I was here in this hospital. It was at the time of Dr Sintayehu. At that time the nurse went to her home and they said to me that I should not go unless the doctor orders and finally they called the doctor and he said give her a five day’s drugs and she should go for the funeral and come back. I went there overnight and attended my son’s funeral and he said [mean to say the doctor] she is good and she will be discharged he was saying to me and this event happened on Tuesday. They he said she should stay here until she is stable and regained her good mentality and I stayed here until sene 14. They killed him at place where he went for work. I passed this temptation here and in those situations they were taking care of me. The sister and also Bekele are very good the doctor also decently he comforted me. It all happened once but you should return to your normal life and continue treatment to be cured he says to me. Even after the funeral I got seriously sick and the sister came and she gave me anti-pain and she secured glucose and she sent my blood to see if there is malaria but there was no any malaria and I recovered soon. I have encountered such challenges and their care is very good.  Mod: In general you say that their care is good?  KI: Yes their care is very good.  Mod: You are close to two years and about to complete treatment..  KI: (Interrupted and talked) … now I again diabetes was found on me and it went up like 418 and again I returned Hidar 2 and discharged Hidar 19. Today also I am measured and they said that it is 365 but I am taking the drugs. The doctor has been suffering with me.  Mod: Was the diabetes known after the treatment for MDR-TB?  KI: It was while I was taking the TB treatment. I completed the TB treatment Tir 4.  Mod: What challenges did you encounter during treatment?  KI: From them there is no any problem I encountered, no any. For the drugs also the doctor has been suffering with me and he pays my transport and also fives me money for lunch.  Mod: Thank you very much for the important information you gave me and in conjunction with the information from others patients I will use the information to describe available service status. I advise you to adhere to doctors’ advice to control the diabetes and if well controlled it is safe.  KI; Ok |
| Respondent-9 | Mod: Thank you for volunteering to be interviewed. How do you describe the social and economic challenge, if any, that you encountered as a result of becoming MDR-TB patient?  KI: Even at my household level it has caused a problem on me.  Mod: Do you mean at level of your family level?  KI: Yes at the level of my family. I mean the attitude they have on me compared to their attitude before it changed and they say go away…Stay there they say to me. I know that it is important to separate utensils but even the way they approach me is does not have any respect. ..They say like ‘you catered this disease unto us from anywhere else you go to’. This disease is not that someone caters from somewhere else but it is caused incidentally. These all things are available let alone others living outside of the home. Other people if they know that we have this disease they even they can discriminate us more.  So as much as possible for this disease it is not good to go back to be with family while on treatment just there in a big center prepared for such patients just there if all that is necessary for the patient is done and treatment given otherwise when we go back to our community even what is the transmissibility ability of the disease we should take into account. While with family we use mask for one or two months we may stop using the masks and start living without the mask. The disease is not something seen as an ordinary disease even for myself I usually get stressed with the disease when I approach others. In fact I should take care for others, I get stressed how much the drugs are very difficult. The drugs are very hard I for example I usually feel pain when I take the drugs.  Mod: In the eyes of the patients how do you describe the nutrition and the transport support that you are getting from this hospital?  KI: About their food, better not to discuss! The food it is very difficult. On the food provided it is very difficult. The food is not such much. If you want to talk to whom can you talk? The food is not good. Before there was like the milk but now all things are stopped. Providing eggs is given. You are given dry injera [Ethiopian traditional food made of a cereal called teff] every morning and at lunch you are given ‘shiro’ [Ethiopan traditional sauce made of pea]. It is not such much.  With this I mean with respect to the food I do not know I mean in the region of Oromia region it very challenging, even there are days when we are denied food. Some days miss some of the daily meal, like breakfast, food as much as breakfast we miss they say that they do not have this and this. The drugs we take the injection in the morning and then at nine we take the drugs how can we take the drugs without having our breakfast. If we have some money we go out and eat some ‘shiro’ or ‘full’ and then take the drugs. Such much problem exists in terms of a problem. In terms of food better not to talk.  Mod: Are you satisfied with the clinical care you are given?  KI: It is nice, I say that it is acceptable.  Mod: Do You get doctors when you need them?  KI: It is challenging overnights, they come through telephone call  Mod: Is there another challenge you want to add?  KI: I as my Ethiopian citizenship what I recommend that here this disease is not an easy disease. Good if the government treats patients in one dedicated center and persons go back to the community after completing their treatment. The other thing is that we may infect others no one knows. What is said at the level of medication which says that it does not transmit if we take drugs, that I do not know but they said us that it stops transmitting after eight months, is that not?, so I say that it is good if the government treats patients in one center at least until patients are non-infectious to the community.  Mod: Thanks for this important information you gave me.  KI: Thanks. |
| Respondent-10 | Mod: Thanks for volunteering to be interviewed? How do you describe the nutrition support that you are getting from this hospital?  KI: The issue of food I do not have any assistant and I need help before they used to give us milk but this time they are not giving us the mild and the drugs are burning me up and how can I ingest the drugs. It burns me up.  Mod: are receiving treatment now as inpatient?  KI: No I am coming from my home. I am living with a relative and get food by going here and there.  Mod: Dot you have your own family.  KI: No I do not have. She was with me before but she died while I was here for treatment.  Mod: Was she died of MDR-TB?  KI: No she was killed by people. I was left empty and now by going to relatives I tery to get food.  Mod. You mean that the food support is not sufficient.  KI: Yes it is not sufficient.  Mod: How do you describe the social and economic burden posed on you by this disease?  KI: On the social life, if a person is sick and he attends the treatment he will get cured. If there is good food and if the drugs are taken properly I also developed this disease as a result of discontinuing treatment.  Mod: were you discontinued TB treatment before?  KI: Yes it was because I discontinued the treatment that it became drug-resistant TB. Now wherever I go I advise many people not to interrupt Tb treatment. Once I developed the disease I tolerate all the burnings I usually eat a piece of sugarcane just to live my body usually I have pain in the joints the doctor said to that as it will disappear to take it easy but it continued to become more severe.  Mod: Joint pains are usually side effects of the drugs.  Mod: How do you describe the clinical care that you are receiving from the hospital?  KI: I have stayed here for long time and it is very good. They also care for me and I want to say God bless. No harm they did and also when I see others’ they are attending them. Those who die is just those whose date of appointment of death is due die otherwise they are good in treating us.  Mod: How do you describe the cleanness of the compound?  KI: The cleanness is good the doctor also tracks that.  Mod: What challenges, if any, have you ever encountered during attending treatment for MDR-TB?  KI: They give us the injection on time and there is no problem. I was treated at Sire and the clinics are not good they annoy you very much. The clinic is Awash melkasa and the managers was very angry and finally they phoned each other and then I got my drugs. They told the clinic that as I have completed the injection I was to continue drugs at the clinic and also as I have cold and I cannot wakeup early in the morning and to cooperate me and it is there that problem exists not here?  Mod: Thanks for your time  KI:  Thanks |
| Respondent-11 | Mod: thanks for volunteering to be part of the interview. how do you describe the social and economic challenge you faced because of your becoming MDR-TB patient?  KI: It transmits you mean when one works with others.  Mod: With whom do you live?  KI: I live at awash in rented house and with my brother. Do you share same room with others?  KI: previously I was sleeping alone in a room but once I took one year drugs I started to share room with others. Until I completed the injection until 9 months I was living alone.  Mod: is the nutrition and transport support that you are getting sufficient?  KI: it is good.  Mod; do you believe that it is sufficient?  KI: Yes it is sufficient.  Mod: What improvements do you want to see in the service?  KI: it is good it is here that we got improvement.  Is there any other challenge that you want to raise/  KI: No any other problem I raise.  Mod: I complete here and thank you for volunteering to be part of this study.  KI: Thanks. |
| Respondent-12 | Mod: Thanks for volunteering to be interviewed. How did you know that you had MDR-TB? Did you take any TB treatment before  KI: Yes, before I was told that I had TB and the six month treatment was ordered for me and the six month treatment I followed and completed it even before I finished the treatment when I was in the sixth month it made me cough. I told them that starting from initial time I have been coughing but they said as the diagnosis was made by x-ray we cannot do anything. Then when I entered the six moth it made me cough very much and I said what is that, should I go for another treatment I talked to the provider and he said ‘anyways let you submit sputum and he said the reason why I kept quiet until today is that initially the disease was not found based on sputum exam he said. Then I submitted the sputum and when I was about three days to complete the six month’s treatment, the sputum was sent to this hospital [she means GeneXpert center]. On day I completed the treatment the sputum result returned and I completed in the morning and I recalled in the afternoon and when I came back they told me that it is drug-resistant TB and you should go to Nazareth.  Mod: Where do you reside?  KI: at Meki town  Mod: okay, what are the social and economic challenges, if any, brought upon you by the disease? How do yyou describe that?  KI: the social problem I think that it is due to wind draft that the disease happened. I was working and I go out early in the morning and go back home in the evening. I have been going to clinics. Then the cough started and after staying for some time it was found to be TB.  Mod: Are you at the hospital as inpatient or are you discharged to treatment follow up center?  KI: I have been sent to the treatment follow up center.  Mod: How do you describe the nutrition and transport support you get from the hospital? Is it enough?  KI: let it be, it is good,…[doesn’t want to talk much about the question]  Mod: now you are being treated for MDR-Tb, other patients will come and get such treatment in same center. So what is the truth about the food and transport support if you are willing to tell me/  KI: The truth is the food is not good for patients, why a patient needs variety of food, why I have been admitted and treated here and food served every day is same type of food, but a patient does not need always same type of food and sometimes delicious food is needed.  Mod: what about the clinical care that you are getting?  KI: the care providers are good.  Mod; Do you get them when you need them?  KI: yes  Mod: how did you get the cleanness of the compound?  KI: the cleanness is somewhat good but around the toilet there is no any cleanness and it was difficult to use the toilet. The rooms are good  Mod: Is there anything that you want to add?  KI: no anymore.  Mod: Thanks. |
| Respondent-13 | Mod; thank you for volunteering to be interviewed. What social and economic challenges did you face because of becoming MDR-TB patient?  KI: No such problem in living with people. In relation to my work I was first year university student and I discontinued my education because of the disease. When I discontinued my education to be treated and cured from the disease only few people provide service with respect. Some of them are not even considering us as human and they do not do what I expected from them. They do not act professionally and some days we wait one person for five to six hours to get the medication.  Mod; is it at the health center?  KI: yes it is and they tell us that as you are victims of the disease we should patiently wait for the drugs but to properly control the disease the best treatment should be given for us as patients to prevent the disease from transmitting. If they abandon us what kind of attitude and response we will have for the community. We should have learnt good things from them. I have been asking about this but there is no any response I get from them. There was one female nurse who provides us the treatment and if she is not there we have to suffer and we need to go to directors’ office and complain, even when we enter the health center compound they run away and even they do not count us as a human being. While knowingly that we should be given medication they run away not to give us the drugs.  Mod: It is fact that you should not be avoided by providers.  KI: Yes we should not be avoided and we should also be examples for the community.. if not the disease even will continue to transmit further. They have better insight on the disease than we are and more than the community but they are doing what is expected of them.  Mod: What about the care you get from this hospital?  KI: here they are good. They are family and they are delivering their responsibilities.  Mod: What about the food you get from here?  KI: When I was in the hospital I used to eat food from my family the food serves by the hospital is not suitable. The important foods like the egg are not served during my stay at the hospital.. also the food is not prepared in the way that s suitable for a patient like increasing the amount of pepper they add in to the sauce.  Mod: How did you see the cleanness of the compound?  KI: Almost it can be said that there is no any cleanness. The toilet is not cleaned regularly and also that all toilets are adjacent to each other. The class itself is cleaned every three days not daily. The windows are not cleaned properly.  Mod: Is there anything that you want to add?  KI: No new thing that I add but if there is possible it is good if you make providers not to discriminate us while we need treatment from them. We did not catch the disease by choice and we unexpectedly became patients so they should understand that reality. No one wants to be patients of such risky disease. As a citizen they should be able to provide the service expected of them.  Mod: Thanks for your time.  KI: Okay |
| Respondent-14 | Mod: thanks for volunteering for the interview. How do you describe the social and economic influence posed on you by the MDR-TB?  KI: The influence id very difficult. The social life you cannot live with others. I know what happened to me.when I first took treatment for the disease it was at Asella hospital. At that time I gone to the hospital with my mother and then when it was said that it is this disease, they brought me to this hospital. Before I came here they came to our home and they said that the disease transmits at the distance of one meter and all the neighbor and all avoided me and that hurt my mentality very much even until now I live alone and that they give me food and you can say that it is a prison for me. It is difficult to be separated from family. It is what God gave me and I did not buy the disease.  Mod: Actually all that breath in air are at risk but still it is not good to avoid patients.  KI: I try to take the maximum care but it is difficult. Now I am on my ninth but still they abandon me. They think of the previous information that they have filled them [health workers educating on household level TB IC]. They still avoid me but I am well now I am working as anyone. After work I go to my class because they still think of the disease.  Mod: Are you living with your family?  KI: No I live with my aunt and my mother is at rural area and my father has died. My friends also do not want to approach me.  Mod: I understand that if they are told about MDR-TB people will discriminate patients?  KI: Yes. *I wish I once sit in the family’s saloon! [emegn*alehu salon metenyat]  Mod: Actually the disease transmits through air but it is not acceptable to discriminate patients.  KI: I also take care for others but as I was khat and cigarette addicted that has made me at risk and I caught the disease while sharing cigarettes with my friends. I started chewing khat in 96 EFY. Last year it made me cough and the six months treatment as soon as I took three days dose it made me to urinate blood and there were white men and one person sent me there and they referred me to this hospital and when I started this treatment it immediately stopped the cough also. Then I totally stopped the khat and the cigarette. Now I am strong than anyone and I have added more strength nowadays. I attend my treatment properly, I eat properly and I have totally stopped the addictions.  Mod: How do you describe the food given by the hospital?  KI: thanks to God that there are also those who do not get such much.  Mod: But what do you wish the reality to be?  KI: For me as I work what I am given supports me a little but those who cannot work let alone being adequate it serves for nothing. There are many mothers of us here for example. They sleep away after taking the drugs. For them the flour is not enough and the milk powder also I have used it it will not serve for more than three days.  Mod: how do you describe the cleanness of the compound?  KI: The cleanness is very comfortable including the rooms. We also live separated by distance.  Mod: Thanks for the detail information. Do you have extra information?  KI: No anymore.  Mod: Thanks |
| Respondent-15 Sex-Male  (HIV/MDR-TB) | Mod: Thanks for allowing us to be interviewed. What are social and economic challenges you faced, if any, as the result of this disease?  KI: I think it may be from hunger and thirst that because I was employed in daily labor work. I work in the deserts.  Mod: Are you continuing the labor work while you are on treatment?  KI: After this disease appears I came here and starter he treatment. Before I used to take treatment for the HIV the drugs all are very much and it became double as there is also the HIV problem. I am admitted here while also using the drugs. There is difficulty it makes me vomit three four times and I am weak. Just by saying that it is a drug and does not bring harm, I just take them.  Mod: how do you describe the nutrition support in the eyes of a patient?  KI: The food is good.  Mod: Is that really the fact you have also been treated being admitted?  KI: Yes it is enough [patient laughs]. The problem is that they do not give on time they cookers quarrel in the kitchen and we are not able to take drugs on time.  Mod: How do you comment the cleanness of the compound, the rooms and the toilets?  KI: In fact the compound is clean. They also change our bed sheets and the blankets and it is good. There is also shower service. We use when we want it.  Mod: What are the things that are challenging for MDR-TB patient in the course of treatment?  KI: after that I do not have any problem. It is good  Mod: Is there anything that you want to see improved?  KI: After discharge I went to Metahara as there is no work here and I go here and there and work labor works so not to beg persons. After discharge unless I do some work how can I get food to eat [implying that the nutrition support he receives is not adequate].  Mod: I think that you have told me all you know as MDR-TB patient. Thank you fro your time.  KI: Okay |
| Respondent-16 | Mod: Thanks for volunteering to be interviewed. What are social and economic challenges posed on you by the disease?  KI: Before I started the treatment I was living with my family and with my friends also. After the disease was known and I started the treatment all have gone to the nearby health center and all have got the treatment and they are healthy, thanks to God and now everything is peace.  Mod: Is there any social problem that the disease brought on you?  KI: No any social problem I encountered.  Mod: There are the so called treatment enablers given for patients like the nutrition and transport cost support. How do you describe that support?  KI: even though the support is not enough, it is a big opportunity to get the drugs for free. Before the drugs were available many of our relatives have died of the disease without getting these drugs. For us getting the drugs is a big opportunity that I think.  **Mod**: Is the food support that you are getting enough?  **KI**: Actually the food is not enough.  **Mod**: What about the transport support?  KI: Even the transport is not enough but I believe that getting the drugs by itself is big chance.  **Mod**: What is the transport cost from here to Metahara?  **KI**: sometimes fifty birr also thirty five they transport us to here. First when I was new to this town I used to come by contract car but now as I am familiar with the town I use public transport.  **Mod**: How did you get the clinical care that you are receiving?  **KI**: The clinical care is very good. It is very good I have also been admitted to this hospital and after I discharged also it is very good. I have also got good improvement after taking the drugs.  **Mod**: how did you see the cleanness of the compound?  **KI**: It is very good.  **Mod**: what are other challenges that you might have come across while on treatment for MDR-TB?  **KI**: For me there is nothing that I consider as much challenge.  **Mod:** Thanks for your time!  **KI**: Okay. |
| Respondent-17 | Mod: Thanks for volunteering and the first thing I bring for discussion is that what are the social and economic challenges that you faced as a result of you being MDR-TB patient?  KI: For me there is no any social challenge that I faced.  Mod: What about work related challenge?  KI:I have stopped working.  Mod: were you too weak to work?  KI: No as I had to attend the treatment I stopped working.  Mod: then if there are dependents upon you who takes care of them?  KI: At that time she was one woman who takes care of them as I had not mix with people I lived alone in separate room and the woman used to prepare food for me and served me well. There is no any challenge from my family but just to safe others from catching this dangerous disease I had to be separated from my family. I separated the utensils I use and my room and started to live alone.  Mod: when your income stopped who will support the family with money?  KI: When my work stopped the one who help the family, I was driving private care,  Mod: Are you married?  KI: No  Mod: So there are no direct dependents?  KI: yes no any I live with my parents.  Mod: How do you describe the nutrition and transport cost provided by the hospital?  KI; I did not take the nutrition support; I only took the drugs and the transport support and used my own food.  Mod: In the eyes of a patient was that enough?  KI: With respect to treatment I think it is enough, since I started the treatment without any difference among them all doctors have been supporting me. Today I have completed the treatment and I talk to you after completing the treatment.  Mod: You mean you have completed the two year treatment?  KI: Yes I have completed treatment and today I have also brought x-ray result and am waiting for release by the doctor. I am left with one appointment only and I say that they all are good in taking care of us. I have been given 100 birr for transport.  Mod: Where do you live?  KI: Here at kebele 10 [Adama town]. 100 birr is not enough for three routes of taxi but the treatment and care service is very good and I do not think that others serve in same way. Starting from the time of Dr Sintayehu I do not say any difference that all of them are very good starting from the beginning.  Mod: Now I see that you have completed the two years treatment for MDR-TB. What are the challenges that you learnt that puts MDR-TB patients in difficult situation and on ways of improving those encounters, if any?  KI: Unless the patient himself interrupts treatment there is no any problem. I for example take the injection and the drugs here every day and return back to home saying goodbye. I have seen many patients dying from the disease and I talk that unless it is due to their own problem, they can get cured from the disease.  Mod: In general you mean that you completed your treatment without any problem?  KI: Yes I both sister and Bekele are here that it is me who completed the treatment without any problem. For the whole eight months I did not miss any dose of the injection and I took the whole injection here in this hospital, even I do not be late for the injection. In fact some of the supports are not sufficient for example the milk is not enough for one month.  Mod: It is very interesting that you are cured from the disease, congratulations and thanks for your time.  KI: Thank you too. |
| Respondent-18 | Mod: Thanks for allowing us for the interview. What are the social and economic challenges brought by the disease?  KI: I used private hospital for many time and even before I mingled up with my families just on my own will I mean. Then thanks to God I completed the treatment and my family also did not make anything on me as they know the case of this disease. We were in Addis Ababa and there were family working as staff and who serve patients. Just they take care of me and also of themselves and in a way that gives me comfort that I completed the treatment.  Mod: So you did not encounter any social problem?  KI: yes with respect to my family there was no any problem that I encountered.  Mod: What about work/  KI: I am not engaged in work still.  Mod: is that because of absence of strength that you were not working?  KI: No it did not hurt my strength so much, I eat and drink well and I take care of myself thanks to God. But as I should finish the treatment I will not work until I complete my treatment.  Mod; Okay that is good decision, how do you describe the nutrition and transport support given by the hospital/  KI: Around this issue there are patients who do not have family and there are also those who have family. The disease selects food and it is your interest if you eat well balanced diet. Otherwise the disease makes your appetite very poor and also when you are given food that is not tasteful it further blocks your appetite. Thanks to God that I eat from my family but those who take the support, they do not eat the food with comfort. It is not what should be served for patients. I prefer tasteful and small meal than much food that has no any taste. I did not take the food, I only drunk the milk, and you are happy if you are given food that is appealing to your eyes.  Mod: What about the clinical care that you are getting from the hospital?  KI: *Wow! It is unparalleled, especially the female nurse, I do not know and ask all patients that they tell you samething. She go all the ups and downs with us. Not common, she phone us and I have two cell phones of her and she phones and encourages us.*  Mod: What are the challenges that an MDR-TB patient can encounter? I mean what things can influence patients taking drugs smoothly?  KI: I think thanks to God, until you get acquainted with the drugs it has bad sense.  Mod; You mean drug side effects/  KI: yes I for example unless I take the drugs and go for sleep I do not have good sense, I may have adopted that pattern. it may be because I get familiar. Thanks to God, the drugs are not same as other drugs. But still it is must to complete the treatment.  Mod: Thanks for your time |

# VERBATIM TRANSCRIPTION OF THE INDEPTH INERVIEW WITH MDR-TB SERVICE PROVIDERS

| Interviewee type | The principal investigator moderated all the audiotaped discussions with in-depth interview participants. Questions are asked by moderator (**Mod**) & answers by respondent or key informant (**KI**). |
| --- | --- |
| 1.Cregiver (C.G. -1)-Clinical Nurse (MDR-TB care provider at TFC)  *C.G* | Are you working now in TB clinic? Yes! How many MDR-TB patients are you supporting with treatment this time? Two! Are you giving them drugs on daily basis? During the intensive phase of the treatment course, all patients take drugs that are swallowed and also injectable. Therefore, each patient should come to the health center to get the injection. The treatment programme is given by DOTS through the whole course of the treatment. But one of the two patients got sick and is now treated at Geda Health center. Why the one patient came to TIC? I mean he was weak and after taking his treatment at my health center for four months as MDR-TB patient he became sick. He failed to gain weight, he also had his own social problem; from the point of food and finance, and in every aspect he has problems. Do you mean that the patient does not have any one to support him?Yes of course, he had a daughter who has been taking care of him, but she died in the Last September (Meskerem). Now the patient is admitted to Geda Health Center. You mean the patient after being transferred to your hospital, re-admitted to the treatment initiating center? Yes he was initiated on treatment at Geda health center and sent to our health center to follow treatment; but now he is readmitted to the treatment initiating center….on the first case, his kidney is failed and moreover he has hypokalemia. Is he old patient? He is 46. How do you describe the nutrition and transportation cost support that MDR-TB patients are getting from the treatment initiating center? Do you feel that it is adequate from what you practically observe from patients attending treatment at your facility? Actually I do not know it, I mean I did not see it, but they describe that they get transportation cost. I guess that it is 100 birr per month but I do not know it. I mean according to what I heard from the patients. Myself I did not see it. Because they come to the treatment initiating center regularly for follow ups. How do they feel you know on the nutrition support they get? They describe that they have support but I do not know that issue. But practically, if we take the example of the patient you described who has social problem including nutrition, does it mean that the nutrition that such patient are getting inadequate? Of course. Where do your patients dwell during the two years treatment period (I mean their housing issue)? Patients usually take daily drugs in their vicinity. My two patients, for example, reside just in the neighborhoods of the health center. If such patients were living far from your health center, how could they attend the lengthy treatment on daily basis? If they were living far, I do not know how they could they attend their treatment, if they were far from health center the transport cost also will be that much, I do not know how they could attend their treatment. But for my two patients, it takes only one route of taxi to reach to the health center.Does it mean that they are in same town? Yes. Do you mean that it is a problem for patients living out of your town to attend daily treatment at health center? Ya! It is obvious. It is very difficult. Doesn’t it mean that coming every day is difficult. If you encounter patients living very far from the health center how do you make sure that they continue taking their medication? If patients are far away from the health center, I mean if it is a serious case it is through supportive treatment, means a person will sign and take the patient’s drugs to apply observation of the daily treatment. it means to apply DOTS. Are community health extension workers practicing daily DOT support for MDR-TB patients? No. no such practice in our situation, other places I do not know. Do you mean that they are not participating? I do not know the other situation, butin may surrounding there is no such practice. The other kebeles I do not know. Now you are providing clinical care for MDR-TB patients. Is your service recognized by your managers? By recognition I mean availability of duty off hours, recognition verbally or through letters? KI: Do you mean for patients? No, for you providers. KII: I could not understand the point. Mod: How do you describe the recognition you get from your facility management and the immediate health office; I mean by such recognition is that, if you get written or verbal appreciation, leave in lieu of extra hour works, etc? KI:I have seen nothing so far; even when you work as a TB focal point and provide care for TB patients, for others even it is not considered as significant contribution. You are expected to prove the drugs for patients as quickly as possible and you are expected to immediately start work in another department. Sometimes providing care for TB patients is not considered as a service. It is considered as simple distribution of drugs to patients. This idea is reflected both by managers and fellow service providers. Therefore early in the morning we are expected to distribute the drugs and join another department to provide other services. This is the situation at our facility. We are said like this ‘simple distribution of drugs you needn’t consider as a big deal’. Thought as the TB patients are coming to “swallow a holly water= ‘tsebelkimesuaynetnegernewu’”  MOD” by that do you mean that it is considered as if TB patients are accompanied when they swallow a holly water as in Ethiopian culture?  KI:definitely!  Mod: is there any concern you can raise regarding laboratory service for TB (MDR-TB) patients? Where do MDR-TB patients give their sputum sample for follow up?  **KI**: For susceptible TB, sputum examination is done at health center. For MDR-TB patients, sputum is collected at treatment initiating center.  **Mod**: Is there any problem you can describe regarding lab related services?  **KI**: No any so far.  **Mod**: How do you describe system’s support (TIC, Province, District, Health facility management) in providing support to you as provider of MDR-TB care and services?  **KI**: The system is good in supporting. I work in a health center located in a town. The town health office TB focal point comes and supervises me. I ask questions and supports on gaps I have and he supports me.  **Mod**: Do you have training on basic TB?  **KI**: Yes  **Mod**: What about on MDR-TB?  **KI**: I have training on MDR-TB  **Mod**: Do you have training on HIV/AIDS?  **KI**: No any.  **Mod**: If you encounter an MDR-TB patient co-infected with HIV, how do you manage him/her?  **KI**: If a TB patient tests positive for HIV, I link the patient to another service site for the HIV treatment. The HIV service is available in another clinic od the facility and I link such patients to that clinic for the HIV related services.  **Mod**: I thank you for info you are giving me and finally how do you describe the overall functionality of the programmatic management of drug-resistant tuberculosis in your locality? What strengths and gaps you perceive as a provider?  **KI:**By the way, tuberculosis, as it is well known is a disease of the poor. I mean, I think you understand me? Mod: Yes. There are many issues like well-ventilated living rooms, there are many, many factors, there are gaps in quickly picking and diagnosing those with two weeks of cough; I mean when we see it in general. Additionally when we see from the side of TB patients, after you have made them, there is problem on providing daily DOTS service.  **Mod:** Do you mean for Generic TB-DOTS as well?  **KI:** Yes, it can be from the perspective of distance, it can be from the perspective of finance; nutritionally there is visible problem. But the provision of drugs is very good. There is enough supplies, there is no incidences of interruption. Now the problemwe have is that one, the problem of the community, for example sometimes patients after starting treatment, that is they start treatment dwelling with someone they have in the town. Some of them do like that. After that they quarrel with that person or family, and they request to go to other places. Do you know why I tell you this, it is just our poverty, it has its own impact.  **Mod**: do you mean that both the patient and someone relative who allows patients to live with them are both poor?  **KI**: Yes, both are poor and it has impact in general.  **Mod**: I want to say thank you again for the valuable information you provided me and we wind up here.  **KI**: Thank you! |
| 2. Caregiver (C.G. -2-Clinical Nurse (MDR-TB care provider at TFC) | **Mod**: Thank you for volunteering to participate in this interview and we start our interview. Has an MDR-TB provider, how many MDR-TB patients do you have currently?  **KI**: Three patients.  **Mod**: how do your three MDR-TB patients attend the continuous daily treatment under DOT, what experience do you have?  **KI**: things that I encounter every day, when I see on these patients from very far distance we sometimes encounter big problems. I mean they are very far.  **Mod**: Do you mean patients live far away from your health facility?  **KI**: Yes, those who catch this disease they are very far, at least they live eight kilometers away from the health center. For thes patients it is difficult to come every day to health center to take daily treatment. I mean this is the main challenge we have. Otherwise there is no much challenge that we have.For this also we are trying to solve the problem by making the patient to rent a living room in the vicinity of the health center and attend the treatment. If the patient has no money to pay for the house rent, we discuss with the management of the health center and try to lessen the patient’s problem. This is the encounters we have for patients on treatment for MDR-TB and it many times troubles us.  **Mod**: well, you raise distance as major factor challenging provision of daily DOT support for MDR-TB patients, and you also rose that MDR-TB patients residing in remote rural areas travel at least 8 kilometers. Yousaid that you are trying to lessen patients’ problems through supporting with covering cost of house rent. What challenges you face while you try to lessen patients travelling long distance and what you want to happen to solve this problem as a general approach?  **KI**: As a general approach, imagine, I have already mentioned it. We sit down with the management of the health center, we discuss with the manager of the health center. If have a fear that the condition seems difficult, for example if the person who assists the MDR-TB patient in helping the patient to take drugs at home is not satisfactory, I mean we go to the level of assisting the patient by renting a house for him. From the health care finance, at least in our surrounding the cost of house fee for rental is not so expensive. It is about two hundred fifty to three hundred birr per month. This cost the patient contributes what he can to contribute. Many times we call the patient’s family; a family closes to the patient, and discuss the issue with the patient’s relatives to help the MDR-TB patient.We also from the health center in the form of the health center management, we have been supporting such patients in this form.  **Mod**: Well, what is the practice existing in involving health extension workers in supporting MDR-TB patients with daily DOT support? What prevailcurrently,are there incidences where they have been participating?  **KI**: Those, on the issue of this disease, they also do not have training. The extensions haven’t taken any training so far. They do not have training. Unless on TB, as they do not have training on this MDR-TB, they are not giving even the other serviceas the case of our “kebele’, I mean as our health center they as they are not giving, it is not satisfactory. It is because of this that we are doing to help MDR-TB patients at our disposal.  **Mod**: How do you describe health extension workers in taking responsibility of DOT support for MDR-TB patients?  **KI**: In reality, in the case of our health center it is very difficult. In the case of our surrounding it is difficult. If you say why, for example, even they are not giving the other TB support. They have taken some training. Even that if you tell them to give it there with sense of ownership, it is very challenging because they do not go to work.  Mod: Do you mean they do not go to office?  **KI**: Yes, usually they do not go to office. Because they do not go to office. In principle of health extension of workers, a health extension worker should permanently live at the health post, because they do not stay the night at the health post, it is very difficult in our case.  **Mod**: well, you said that you are providing some social support to your MDR-TB patients. The other issue is nutrition support they are getting from the system. Do you think that the current nutrition support provided for MDR-TB patients is adequate?  **KI**: The nutrition support that patients are getting is not adequate, nothing there. Even I have not ever seen it. We also haven’t ever made any support as in our case.  **Mod**: How do patients describe the nutrition support they are getting from the hospital?  **KI**: what they get from the hospital. When they first stay at hospital for about two months. When they are treated at the hospital, patients tell us that, they ushered them properly. There is food and advice also and other things they tell us that it is full. When after two months,when they are sent to our health center, the condition there is good that they tell us.  **Mod**: After they are sent to you, how do patients get the so called nutrition support? How do patients get the nutrition support from the hospital?  **KI**: In our disposal no nutrition supports.  **Mod**: No nutrition support at your setup, but don’t they get nutrition support from hospital?  **KI**: They say that during their stay at hospital for two months they state that the nutrition support at the hospital is good, they tell us when they come to our health center. When they are admitted and treated they tell us that they get good nutrition and after two months, when they come to our health center, they usually can tell us that nutrition support is available. But we tell them that that service is not available at our disposal.  **Mod**: Your MDR-TB patients stay with you for a long time, what experiences and challenges have you encountered during your long stay with your MDR-TB patients and how do you overcome such challenges?  **KI**: The experience I encountered, for example, is with respect to drugs. They say that the drugs burn them up, they also complain that the drugs change the color of their urine for the first time. After that since I took the training, that experience having known that such patient experience is due to drugs, for example that the drugs burn me up means that it causes gastritis, I give that person drugs for gastritis and then observe when the patient gets relief from that gastritis. At first it was challenging me thinking whether the drugs could cause side effects or not. This is the experience I encountered and even sometimes they say pain of jointsand this thing I do not know what it is and the drug like cycloserine, when you discontinue, it disappears. With those the TICs will make contacts, we phone to Doctors and when we tell them that patients have such and such problems, they tell us to stop such and such drugs for some days, so at that time injective, it is injection that has such joint pain and tell us to stop and when we stop them, it brings change.  **Mod**: Well, the other issue is that recognition for the service you provide.Do you feel that management of your health center or management or the district health office recognize the service you provide on MDR-TB?  **KI**: Regarding the service I provide in the TB clinic, every day I start working at the unit starting at six o’clock. I also reside around the health center and I enter there around six o’clock in the morning.Up to like seven thirty, for all types of TB taking drugs I send them back up to that time. After that other workers come to work at eight thirty. I will not go to work at eight thirty. Because I start working around six in the morning, I go home and start normal working hour at about nine thirty towards the TB service center. For that as I start working early in the morning the health center manager assists me for that. Saying like ‘why you fail to come at eight thirty’, the health center does not bother me with meeting the attendance time.  **Mod**: Well, but you are given tasks to accomplish besides the service you provide on tuberculosis? How do you describe the support of the district health office on the programme, how do you feel on their collaborative support?  **KI**: With respect of the district health office, when I take the district health office, the support only when they need ‘slides’ and reports only that we communicate each other. Thoseat the district, especially those at the zone (province), since I came to this service unit, it is about eight months, before I was not there. During this eight months, no one came from zone for support, even if you cannot come in person you may call and ask the situation. No one do that. I have not ever heard of such. There is gap regarding zone. The woreda (district) calls sometimes. They need reports monthly. For follow ups. They tell us that we should not forget patient follow up services and they call. No one come down to the health center and who identify the data and the things available. I raise that in this regard they have gaps.  **Mod**: From your saying, I understand that, all that the district health office do is asking for reports and not coming to health center to supervise you and monitor your work?  **KI**: Yes. Regarding the issue of MDR-TB, I don’t think that the district health office knows the problem.  **Mod**: Do you mean that the district health office doesn’t know presence of MDR-TRB?  **KI**: No, actually they know presence of the MDR-TB as a disease. But having the ownership, unless it is for the other types of TB, for the MDR-TB; how many patients do you have, where are the MDR-TB patients, until this time, even there is no any report that they took on MDR-TB unless it is on other types of TB. Do you understand what I am saying? **Mod**: Yes I do. KI: But the zone health office, during the past eight month, in person, let alone in person even by phone they haven’t done anything. I mean we have not made any contact so far.  **Mod**: Do the TIC hospital providers come to health center?  **KI**: The TICs are very good. The TIC and our, the TFC it is good. I mean good relation between the two. If there is drug shortage we call, if there is the patient complains side effects we tell them that the patient complains of this and this things and then they tell us saying do this and that things. They also come every month. They bring drugs for us. We also communicate with them through phone. In general, I mean, it is by the help of the TIC we are solving problems we encounter during patient treatment.  **Mod**: Well,do you have training on basic TB?  **KI**: yes.  **Mod**: on MDR-TB?  **KI**: Yes.  **Mod**: what about on ART?  **KI**: No on ART.  **Mod**: If you encounter TB/HIV co-infected patient, how do you manage?  KI: If I encounter TB/HIV co-infected patient, there are those trained on ART.  Mod: Do You have ART center?  **KI**: Yes.**Mod**: So do you refer the patient for ART? **KI**: Yes. **Mod**: Does it mean that cannot prescribe ART drugs? **KI**: Yes I cannot.  Mod: You need lab service for MDR-TB either to diagnosis suspects or to follow those on treatment. How do you describe the available lab service, and the elation you have with regional labs?  **KI**: Regional laboratory, now for the past four months, the regional lab has stopped providing service. We have also taken some training here. They say that the equipment is failed and they mention that they could not get the equipment even by going to South Africa. Not only that, there is shortage regarding laboratory. I think that it is a national problem. For example, as in case of our health center, we have only one. Plus, the government, I think that I have opened the so called postal system. Everything goes through postal system. It is packed and it can be viral, it can be CD4 and also sputum. These all, they said, that they should be transported through post office. This thing for the labs, if we see as in case of our labs, when we see the regional lab, the relationship between them, usually for the facility laboratory, it is the regional lab that provides training. But here when the postal system, takes the sample off the facility lab and transport it to regional lab,the lab there, I don’t know that the awareness given may not be adequate; they do not collect, label and pack samples properly. The way samples given to postal system is not good. I think that if the sample is lost in between they want to go by themselves and get per-diem, when I see the situation. When they remain at the health center, because they do not get perdiem, previously because they get perdiem when they transport samples in person, currently creation of sample transport through the post system on the lab persons, it is decreasing their work morale. This is what I observe as our health center. The lab works just remaining in the lab, samples of viral, CD4 and sputum of MDR-TB suspect, because they say it will not go, I mean it is sent with the post office and this makes the facility lab person not to get that service the per-diem, when he packs and delivers the samples they do not do it properly. Then when the post office takes the samples to regional labs, those information mentioned can be lacking.  **Mod**: Well, Finally is there any thing that I did not raise that you wish raise for discussion?  **KI**: In general regarding the disease we have been discussing over, in depth all those concerned, as we rose above, the zone and the district also, because they have gaps, the message I want to pass on this good if it is done in more strongly. It is called that MDR-TB is changed to XDR-TB, recently as I heard, around Bishoftu, they say that there is around ten patients of this XDR-TB. For this, if you say from where the gap starts, if the zone does not go down and support and push woredas (districts), and the district in turn go to health facilities and push, it is a big gap because the condition is going to XDR-TB.So I say that all concerned shall support the service.  **Mod**: Much thanks for participation, we rap up here  **KI**: Ok. |
| 3. Caregiver (C.G. -3-Clinical Nurse (MDR-TB care provider at TFC) | **Mod:** Thanks for volunteering to participate in this interview.How many patients do you have currently?  **KI**: Now there are three patients at my disposal. Two of them attend follow ups at Bishoftu hospital and the other one her follow up is at this hospital. Normally the problem on MDR-TB is, there are many problems we are facing. Firstly this disease is the disease of persons with very high problem. For example the one patient who attends her follow ups at this hospital, she is called Asnaku Eshetu. She lives in the town. She lives alone. No one to support her. Her mother and her father, they have no income. They are poor. When she first started treatment for TB, she was treated for normal TB with first-line drugs just at my hospital. While she was taking drugs at my facility and when she gave her sputum at fifth month, it became positive. Then I sent her to this area and they said it was said MDR-TB. Then she directly came here and for one month she took the drugs here. Then she came back to me. When she came back she lives alone, she has nothing to eat and also she said that she has no one to support her. She comes and as she takes daily DOT, I ask her daily problems. She suffers much. I have also gone and see her home. When I go there she has nothing to eat and in general she lives in different situation. She has brothers but they could not understand her. I have also talked with her brothers about the prevailing situation. At that time she is also psychologically, patients taking these drugs usually their psychology is usually it will not continue to be the same usually. She had relations with her brothers and they have a different perception about her behavior. Usually they say like why I caught this disease and why I take such drugs. Their awareness is low. As their psychology deteriorates in relation to that, she is facing much problem.  Mod: You mentioned that the patient is poor and also she has no one to support her. KI; yes. Mod: how do you perceive, is the nutrition support that the MDR-TB patients are getting monthly after they are linked to treatment follow up centers adequate?  **KI**: Normally, I cannot say much. I do not know the nutrition support given to them because I havenot seen it.  **Mod**: What do you hear from patients about the nutrition support they get? What do you understand from patients?  KI: Usually I ask, and what we understand is, I ask them like ‘are you given nutrition support at TIC’. She says yes but it is not enough, according to my perception it is not enough.  Mod: How far do the three MDR-TB patients dwell away from your health center?  KI: In our case as they are in town it is not too far.  Mod: Are the three in town?  KI: Yes.  Mod: Are you providing treatment under daily DOT support?  KI: Yes.  Mod: If it were the case that some patients come far away from the health center, how do you ensure continuity of the daily DOT treatment?  KI: for example, the one who follows her treatment at this hospital, she is on her 15^th^ month. Until that she has been in the town to attend her treatment. But now her mother lives in rural area and it is not very far. Currently she is living there. She is coming daily from rural area. When I ask her as to why she go to rural area, she told me saying ‘I have no one to assist me and also as this time is a harvest time, I should go there and help them’. She said that it is must.  Mod: can she daily come and take her drugs?  KI: Yea! It created problem on that. Actually it is a problem. So far she has been properly attending her drugs. Having seen that, I have giving her drugs every three days to take at home.  Mod: can she differentiate the different second-line drugs?  **KI**: Yes. She knows more than me. There are four types of drugs. For four of them, she knows the side effects she can encounter when taking each of them. She differentiates and knows how many tablets of each to take because once an individual takes similar drugs for one month he can differentiate such drugs.  **Mod**: Just on this issue how can you describe the participation of health extension workers on supporting daily DOT in your area?  **KI**: correct! In case of our ‘kebele’ the participation of health extension workers, it is not adequate. On MDR-TB the public knows nothing. Not only the public but the provider also does not have adequate insight. Because training is also not given to all providers, for providers working in our health center I have made them aware on the drugs and the amount taken. But initially all fear when it is said MDR-TB. Currently there is some improvement.  Mod: Do you feed supported by the management of health center and the immediate health office on the MDR-TB services you provide? For example, are you exempted from other activities because of the extra ours you spend on MDR-TB?  KI: Yes! On health center management and woreda (district) regarding MDR-TB you can say there is nothing. Health center head, for example, does not know the dangers of MDR-TB, the type of drugs that patients are taking. Because I think that they also do not have trainings. They simply fear when they hear the issue of MDR-TB but they do not give much credit to the service given.  Mod: So as per the perception in your health center, whose responsibility is the problem of MDR-TB?  KI: Because it is just from the higher level. On this issue if we discuss the problem with health center head it is one thing. But health center head on the issue of MDR-TB they do not have much concern. Do not give priority to the disease. I believe that if managers give priority to the disease, many problems will be solved.  Mod: I understand that you mention that you are not getting continuous support from health centers and district health offices. Is that right?  KI: yes. Even the woreda (distict0 health office regarding the MDR-TB no one asks us about the issue.  **Mod**: How do you describe the laboratory service on MDR TB? It can be for evaluation of MDR-TB suspects or for follow up of those on treatment?  **KI**: In that issue. There is follow up service both for first-line patients and MDR-TB patients. For MDR-TB their follow up is just here at hospital. Just for drugs they come every month and they give sputum. At our health center for normal TB at month two, month five and month six there is laboratory at our setup. But for MDR-TB that is called presumptive TB, we send them to this hospital.  **Mod**: Is it going well?  **KI**: Yes. Up to now the sputum is sent through post office and so far the situation at Modjo health center is good.  **Mod**: Is the post office system of sample referral functional?  **KI**: Yes it works up to know.  Mod: do you have training on basic TB?  KI: yes  Mod: do you have training on MDR-TB?  KI: Yes  Mod: do you have training on ART?  KI: No  Mod: If you don’t have training on ART, how can you manage MDR-TB patients co-infected with HIV?  KI: Normally TB and TB/HIV all patients registered for TB treatment are screened for HIV. From those tested if I get HIV positives I send them to ART clinics.  Mod: Well, apart from all issues I raised so far is there anything that you would like to be discussed?  KI: According to my believe given all the efforts done so far, the relation with patients especially the TFCs it is important to strengthen patient follow ups. For example there is a saying that if the patient returns after one month treatment interruption, it is good if monthly follow up is done from hospital at least by phoning the MDR-TB patient. I recommend more connection between TIC and TFC that that exists currently. Now currently there is review meeting every two month (between TIC&TFC). This is good. There is Debrezeit. It is not this much. I think they do not have much patient. It is good if they do same as this TIC.  Mod: You need that for discussion and joint action on available problems/  KI: yes  MOD: Do you have any issue to raise regarding drugs like availability, storage etc?  KI: No problem so far.  Mod: In grereral these are points I want to raise and we finish here and thank you for participation.  KI: Thank you. |
| 4. Caregiver (C.G. -4-Clinical Nurse (MDR-TB care provider at TFC)  Sex: Female | **Mod:** How do you describe the conditions around providing continuous DOT support for MDR-TB patients treated at your health facility?  **KI**: Concerning the continuous DOT support for patients, it is given correctly at our health center. But it is considered as responsibility of only one person.I mean as in case f our health center. First when the problem of MDR-TB came there was huge problem. If I am not present in the health center there were condition when drugs are not given for patients. It is from the prevailing fear a little bit the providers are not aware about TB.  Mod: Do you mean that providers do not have awareness?  KI: Yes! They are not aware but apart from that there is condition whereby there is continuous improvement. Currently there is good situation prevailing. It seems like this regarding DOT support.  Mod: where do your patients live? How far are they from your health center?  KI: Now do you know what it is. There are patients taking at health extension workers.  Mod: Do health extension workers support you with MDR-TB?  KI: No it is for normal TB  Mod; how many MDR-TB patients you have currently?  KI: Two  Mod: where do they live?  KI: Just they live at Sagure.  Mod: You mean they are not far from health center?  KI: Yes. They are living in the surrounding and they come daily and take their medication.  Mod: If your patients were living far from the health center, how could you manage/ Do you expect any problem?  KI: I haven’t encountered any patient from rural area so far. For example one of my patients was from rural area. Her family lives in rural area. It is much kilometer far but she comes for injection every day and takes her drugs every fifteen minutes. Because of this the drug itself makes them weak and brings headache and other things. She lives there by renting house.  Mod: Who rents the house for her?  KI; The family rents the house for her. Therefore there was a condition whereby she could follow here treatment by renting house in the vicinity she is taking drugs there now.  MOD: What can happen if her family could not rent a house for her?  KI: Now I should report to the worea (district). MDR-TB is very dangerous. MDRis not something that we take at ease. It is national issue it is too much. Now as I haven’t encountered such problem they accept all recommendations I tell them.  Mod: In some facilitiespatients come more than eight kilometers away. If you encounter such problem, how do you manage?  **KI**: I think that I should discuss with woreda health office and solve the problem. Such economic problem it is all of ours problem that I think. So far there is one normal TB case that I want to tell you. She did not have any money. In that there was a condition whereby the staff contributed money and made the patient to complete her treatment.  **Mod**; Is it you who mobilized the staff?  **KI**: Yes. She had some mental problem. When the staff receivesalary we rented house with some money and covered her expenses and enabled the patient to complete her treatment.this is for normal TB. If this case encounters I should notify the woreda keeping the steps and I believe we can do much. Otherwise I don’t think that we neglect such cases.  **Mod**: Similar to same issues how do you describe adequacy of the nutrition support allocated for patients after they are linked to treatment follow up centers?  **KI**: on the issue of nutrition for those need nutrition we give them plamynut but there is problem on nutrition support. The problem is very huge. Nutrition in relation to MDR-TB, they do not have anything. They are poor. Therefore this thing is very difficult. There are many complaints on that.  **Mod**: What do you feel regarding the recognition you get from your health center management and from your immediate health management on the services you provide on MDR-TB? Do you feel supported?  **KI**: on the support I need they usually support me. The health center head when I tell him that I need this and this he usually help me with that. I am supported with what capacity allows.  Mod: How do you describe your relation with TICs?  **KI**: We meet monthly. They meet with patients monthly. They also give us feedback. We also get feedback. So our communication is good.  **Mod**: What things do you want to raise on lab service you are getting on MDR-TB, that is it can be for diagnosis of suspects or for follow up services?  **KI**: With regards to laboratory, they usually do as per our request. Sometimes they complain shortage of reagents. Sometimes there is lack of giving attention to the issue that is problem of negligence and there is also problem in meeting what is expected of them.  **Mod**: If you send MDR-TB suspects for evaluation do you get prompt response?  **KI**: Yes, if we need the GeneXpert they usually call the post office and send and it is good.  **Mod**: Does the post office works well in your case?  **KI**: Yes, it works.  **Mod**: Well, I am winding up my discussion points and finally I invite you to raise anything that you like to be discussed regarding the MDR-TB issue.  **KI**: The things that I want to add is that in one health center only one person should not be trained on MDR-TB. As a country MDR-TB is a serious thing. It is a disease that causes huge crisis on the society. Therefore what I say to be stressed on this issue is that not only the health center but the zone (province) itself and Oromia itself good if the training is expanded. In one health center more than three to four providers should be trained. For example a person may go for vacation, may go for maternity leave. But the issue is something that you follow daily. Therefore the task should not be left only for one person.  **Mod**: Are you assigned alone now?  **KI**: Yes I am alone now. If I am absent, the service is absent. I work including Saturdays and Sundays. So good if two three persons are trained and assigned to work with responsibility. If the problem is given due attention. I believe that the problem can be solved in that way. What I raise as a problem are these things.  **Mod**: Thank you very much for you are volunteering to participate in the research and I wrap up here. |
| 5. Caregiver (C.G. -5-MDR-TB Physician | **Mod:** As a clinical mentor, what is your perception and level of confidence on strict continuity of DOT support for MDR-TB patients you link to treatment follow up centers for the two years?  **KI**: I can speak in full confidence on that. This is because once we send patients from here we have communication with providers at TFC and we confirm whether patients have reached to TFC. When we take patients there we will also take medications for the patients. Plus all patients have my cell phone. They personally phone to me. If the provider is absent, the patient himself calls me the patient himself calls me. Today providers are absent; today my drugs are stock out they tell me. Even before providers themselves phone me that they phone. So they tell me if they miss a drug. The providers gave me painful injection they tell me, it bleeds they say even. Therefore I do not have any hesitation on daily DOT. If there is someone who does not take drugs they tell me in two to three days. Not only this when we call for catchment area meeting, for focal persons we give them good awareness. If there is something beyond their capacity, I mean if the patient refuses if adherence problem is encountered and we agree that if a patient misses one day dose we have to know on the second day. If they encounter any problem they will tell us. Apart from those patientsthemselvescommunicate us. All available patients have my cell phone and they call me.  **Mod**: You mean that you have handed over your cell phone to all patients?  **KI**: Yes. They all have my phone and they call me. Moreover the providers in our case,may be that is the main reason for the low rate of lost to follow ups in our case. The patient communicates us; they (provider at TFC) also communicate us. Things we get when we go for mentorship, there are things that we cross check. We get patients there, there are things we check from other staff. Therefore I am confident thatthey take drugs.  **Mod**: How do you transport MDR-TB patients from TIC to TFC?  **KI**: When they go from here to TFC, we cannot say hundred percent. There are patients transported by ambulance. But when there is deficiency in ambulance, they go by public transport. We advise them on what we can advise them. They use the face mask, which is the surgical mask. It looks like this. But almost all ambulance is not available most of the time.  **Mod**: Thank you, the next question I forward for discussion with you is that things of patient treatment enablers or incentives like covering cost of transportation, nutrition support. How do you describe that type of support for MDR-TB patients when patients are a TIC and also when they are treated at TFC?  **KI**: At our disposal, for me there is good thing that is available. I mean to tell you the truth. This is because since I came here, it is more than a year; when a patient comes by transport the patient brings transport receipt. Double amount of what is in the receipt is given to the patient(the KI meant that round trip transport cost is covered).For patients in same town it is not transportation fee that is paid as for patients coming from far. There is the so called one hundred birr. I have seen the breakdown myself. I have myself downloaded and seen it. They get their nutrition support. They gethundred percent. But when we say this it has many risks for us. It has many debates. At finance sometimes the transportation breakdown we submitted like transportation for example, we propose that we give them round trip that is double and when we submit there are incidences whey they pay only single trip. There are times when they do not volunteer to pay. Many times they are nurses who handle the payment for patients. Especially this time because they say that they do not want to go. They are nurses who pay. The finances say that they do not want to go. Therefore we have debates on budget. They say that the budget has been used up, that means even when the budget is actually available. Normally we have, we retain the breakdown paper what amount we have used everything but still if they want they say that the budget is not available means used up, other time they say that they will not go and make the payment. We do not have cashier. This is the problem. Otherwise by making every fight there is a situation whereby patients are getting. Even in one step for example the residence of one patient is ‘X’ residence area for example if we request round trip cost and if they pay only single trip transport cost only, on next month we wait and make additions in lieu of the currently not reimbursed transport cost and make the payment on next month.  **Mod**: As you mentioned I understand that, it seems the way the resource used matters more than the issue of its adequacy. Is that right?  **KI**: It is not the absence of money but the way it is utilized. In the finance class they challenge us very much. But still the patient is beneficiary hundred percent. But for us it is very risky because it is not our professionally responsibility. Even when we go and ask them, they mistreat us saying “your patients”. They say “your patients” considering as if the patients are our family members. There is such situation.  **Mod**: You mean that the finance consider as if it is only your team’s responsibility.  **KI**: Yes  **Mod**: What are the practical challenges you face in management of drug related side effects?  **KI**: The practical challenges, firstly the side effects the chance to detect them is very difficult. Many of them are known through laboratory diagnosis like for example electrolyte it is very killer. You may not detect it symptomatically I mean until it is in advanced stage. These things especially at TFC they are challenging. They cannot detect it for us promptly. I can raise one patient as an example. She developed. She developed hypocalcemia and what she did was as she has money she went to private wing clinic. When she go there they said that it is hypertension and gave her anti-hypertensive drugs. She is seizing, it is hypocalcemia tetani. They made her start nefidipine. She is 21 years old. At that time I had called catchment area meeting. Then when we talk there they told me that the patient was diagnosed with hypertension and she is admitted they told me. Then I said no this cannot be hypertension and I asked them to bring the patient on the next day and I said that I admit and follow her. Then she is admitted. When we follow her BP, BP is normal. In that way she has developed hypokalemic tetani. When we measure it, it was severe hypocalcemia with hypokalemia and hypomagnesemia. Therefore as the symptomatic identification of these side effects is challenging especially at TFC, it is very difficult to detect. Not only there at our disposal also electrolyte is not done.  **Mod**: do you mean the service is not available?  **KI**: Yes. Especially these days nothing is done.At one time there is reagent shortage. The other time lab technicians are not cooperative. We take the sample and they tell us that the sample has expired. But most of the time. Sometimes they say that the machine is not functional. More than anything else is the reagent.The reagent. The machine. Lab technicians failing to be cooperative addsup to create very huge problem especially on electrolyte.  **Mod**: What can you say about availability of ancillary drugs use need to treat these adverse drug reactions?  **KI**: Ancillary drugs what we do at our hospital is that with the main hospital pharmacy we together as if it were owns issue we made agreement and we take from them and give to patients. Theyget free. Almost for medications available at our center they get for free the medications.  **Mod**: so you mean that you do not have problems with access to ancillary drugs?  **KI**: Yes. That we discuss with pharmacy and we give them and they use it. So there is no problem.  **Mod**: Then when you think as a physician there was something you mentioned earlier you said that others say your patients, your patients. How do you think the recognition of your of your service?  **KI**: I can say they don’t recognize it.  **Mod**: Is that?  **KI**: Yes. They don’t recognize it. Let alone this what makes me surprised is that the so called finance, the so called medical director, the so called provostand the so called CEO, they do not have any concern for this issue. At our hospital, for example, a car was allocated and given to us for TB for mentoring and for doing other important things when exists. They took it from us. They said to us why do you come. When we go there for example, the reason why we come to hospital *(KI means from MDR-TB treatment center to hospital)*is for example there are many things that we facilitate. The physician assigned there is facilitator of logistic issues at the same time. If water and electricity is discontinued it is the physician there who fights. Therefore we are logistics, finance as it is we who runs the financial payment. This is because they do not support us. We are pharmacists-it is we who receives and dispenses drugs.We are laboratory technologists as it is we who collects laboratory samples. But the medical director says why you come for fear that we are coming to take the car assigned for us.  **Mod**: In general how do you conclude the support you get from hospital management, province and the town health office?  **KI**: Around hospital management. No question it is very poor. It is very poor. We have been debating repeatedly. I have criticized them repeatedly. I have notified to Oromia region health bureau and the HEALTB organization repeatedly. It is very poor. They do not understand the issue as you do. They have very low level of perception on MDR-TB.  **Mod**: So you mean that MDR-TB is considered as the problem of the physician and not the problem of the system as a whole?  **KI**: Yes.  **Mod**: Well. How do you describe the lab service you have currently?  **KI**: on laboratory normally we do what the guideline states. We do all baseline investigations. And then we have follow up investigations. Laboratory including probably smear and culture organ function tests and so on we perform. But sometimes when we go to issue of culture there is delayance sometimes. There is incidents when they do not volunteer to give back culture result. There are debates with regional labos like they say “we have delivered the results” andthe like. Result delays and it looks like this. When we cometo serology like chemistry for example, they say that the machine is not functional. Moreover there are times when we collect and store samples aside and the sample expireas there is no one who processes the sample for us.In general the prevailing situation seems fair for patients but there are problems.  **Mod**: You mean that you are treating patients with enduring all prevailing challenges.  **KI**: Yes. It would have been more encouraging if the unit [KI refers to MDR-TB unit] has its own lab unit. A dedicated lab who works for the unit only. If I take a sample today, for example, anyone who wants to process processes the sample and anyone who is not volunteer rejects the lab request we make. Or the laboratory person asks payment and says that he/she is not volunteer to process unless he/she is paid. To tell you the truth it is by paying them that we know get their support.  **Mod**: Do your team providing MDR-TB care have training on ART?  **KI**: The do not have.  **Mod**: How do you manage your MDR-TB patients co-infected with HIV?  **KI**: In our setting as the hospital and MDR-TB center is not in same place we normally update ourselves as physicians. The nurses are also with us. Their main follow ups for the ART clinic is separate that they go to.But still we are in touch with ART clinics as we meet in person also. The other issue we physicians update ourselves and manage patients. Otherwise their main follow up center is the ART clinic. If there is problem we communicate.  Mod: Do you think that it would be good if there is an ART service center in the same compound with the MDR-TB treatment center?  **KI**: It is very good. It would have been excellent. It is not questionable. This is because we had one patient who died at 17^th^month of treatment which was surprising. The patient was found fallen down on the street. The patient has been on ART for seven years. Then they brought him and when I saw him CD4 was forty. It was ART failure.The patient has been on MDR-TB treatment for seventeen month and have converted culture. The patient was left with three month or so to cure. The patient was found comatouse. The ART failure was not diagnosed. Therefore I believe that had there was ART there it could have improved many things.  **Mod**: Do you follow patients after they have completed treatment and released from treatment?  **KI**: After completion yes for two years as it is dictated by the guideline.  **Mod**: Finally is there any issue that you like would have been raised concerning any thing around management of drug-resistant tuberculosis?  **KI**: The concern I think of is that the awareness is low. Especially around the hospital management including medical director. The awareness they do not understand the situation prevailing there. So they have to know it. They should understand us including the finance. The other concerning zones, we do not need them very often. We meet if patients are lost to follow up and on catchment area meetings.  **Mod**: Otherwise you remarks that patient management is your task?  **KI**: Let alone patient management the physician in our hospital is not only managing patients. If there is no water it is the physician who fights, if there is no light it is the physician who fights. Purchase of coal, oxygen is not available many times and the issue of oxygen procurement is the concern of the physician. Going to pharmacy to beg for drugs is done by the physician. We bring drugs. It is we physicians who transports laboratory samples, it is the physician and the nurse who is responsible for patient financial payment. Thus it is a very huge load for physicians. It is a headache. On top of this the perception they have is challenging. They perceive you in the wrong way and it is difficult.  **Mod**: We wrap up here and thank you. |
| 6. Caregiver (C.G. -6-MDRTB care provider-  Nurse | Mod: I am MengistuKenea and thank you for volunteering to participate in the interview. It is known that your  patients are linked to catchment treatment follow up centers after stabilization at your TICs. What is your  confidence on the continuity of the daily DOT support for patients linked to  KI:Thanks. In fact, at our hospital for patients we start treatment after they are initiated on treatment for about one  week here until they are stabilized and they attain the adherence needed we make them stay here. After this  they are linked to a treatment follow up center found nearby to their usual residence area. Thus after they go  there we are sure on their continuing their treatment because we have contacts through telephone call with  health care providers working there and we discuss on the condition of patients out there. Secondly for  those health care providers when we call them to the catchment area meeting, the providers from the  treatment follow up center gives us updates on the current and fresh status of each of their patients and  make explanations. Because of this we are sure that they are properly taking their drugs because the reason  why confident fully is I mean are based on the culture result they have. They come every month and attend  their follow ups we do sputum and blood examination for them. It is based on that their result that we become  sure that they are taking their drugs properly. The way we confirm for absence of any problem is that they  continue to show improvement from time to time. It is based on their result that we make sure for their taking  drugs properly. Apart from that we go mentorship every month. After we go there we verify whether patients  are taking their drugs properly by seeking to their documents properly. Through such and such means it means that we are fully confident that our patients are currently on good condition and their result is those  beyond two month, at month three all convert culture. Because they convert culture two month after starting  drugs and they convert on the third month, we make sure that they are taking their drugs properly.  Mod: Thus you mean that there is no hole in the continuum of care that the patient is getting from the collaboration  between hospital and health center?  KI: Yes there is no any gap>  Mod:For our patients there are treatment enables through covering transport cost and nutrition support. How do  you describe the condition around that?  KI:So far when it was started initially it was given to them in the form of cash. After that those concerned officials  discussed on the issue and they recommend that giving cash is not a good practice as they may use the  money for another purpose. As a result it was decided to be changed in in-kind or in the form of nutrition  support. That issue we told to our patients and we convinced them and also we made our providers at the  follow up centers to tell and convince patients. Being convinced this time it has been long time since that they  are being given in the form of food. That when they come every month when they come for follow ups, we  give them that. Apart from that they are paid transport money and they are also given pocket money.  Therefor there is no any problem on that currently. All our being convinced all are taking the nutrition at this  time.  Mod:Related to same issue don’t patients complain transportation of the food items to their homes?  KI: In fact there are complaints. For example for those living very far transporting the nutrition items, it was initially  that they were complaining transporting the nutrition items. They were saying that they could not transport  that weight and they complained transportation problem. At this time there is no such complaint. Because  they know that what they take today will benefit them tomorrow they take it home. There are also some who are poor those who do not have families who do not have supporters. There are patients depend on this  support. Therefore at this time if there is even transportation problem, they are taking it home enduring its  transportation problem.  Mod: Thanks. You explained well the issue of treatment enablers. The other discussion point is the laboratory.  Laboratory is at the center of the service you give both to diagnosis MDR-TB and to provide follow up  services. Would you please explain for me the condition of laboratory on the service you provide?  KI: Concerning laboratory, in fact there is shortage of human power on laboratory. Now the professionals we bring  is, we bring them from hospital. When every month our patients come for follow ups we make them to collect  sample. Sometimes when there is shortage of human power and when they cannot come here we ourselves  collect the sample. But after reaching there they properly process it. But this time there are some reagents  problem, there is also no maintenance. For example serum electrolyte is a very necessary thing here our  patients taking MDR-TB treatment these things this days there is problem of reagents. Machines sometimes  there is incidents when they become non-functional. On these things it is good if there are ways to maintain  machines and to make reagents available. Apart from that on the laboratory there is no big problem.  Mod: From the register it is seen that for patients those have been getting lab follow up services previously, there  is no follow up done and registered recently. I understand that this may be associated with the absence of  maintenance for machine failed to function?  KI: Yes. It is right. In fact the machine is available but sometimes when a problem is encountered, the machines  may failure, and up to now as the machines fail many of our patients may not get follow up laboratory  services according to the standard.  Mod: Here you are clinical care provider at the TFC. Do you feel supported by the hospital management and the  health system in general?  KI:Definitely. The hospital is doing all what is permitted by its capacity. They are doing all that they should do for  us. For example medications, ancillary drugs for like the side effects it is the hospital up to now that supports  us with the drugs. In fact there are ancillary drugs that are delivered through partners. But even as those may  not provide all the drugs we want the hospital is supporting us. In fact they support us and there is no  exaggerated problem that we have.  Mod: How do you describe the involvement of the health offices?  KI:Yes, the zones in fact they come closely some things if there are problems for example if there is any problem  with the patient, if there are problems at woredas (districts), it is with the zone that we report to the zone and  the zone if the patient discontinues treatment, what is the problem they follow and solve the problem. They  contact with the woredas and something they provide us with fruitful feedback and we have good relation.  With the town administration also we meet sometimes, we meet on catchment area meetings and jointly  discuss the prevailing situation, and if there is problem we devise means by which we solve jointly.  Mod: The next and probably the last discussion point I have is that, currently you have MDR-TB patients coinfected  with HIV. Such patients need treatment for both diseases. How do you manage them? Is there any  one here with training on ART?  KI:Yes, basically two of us nurses who work here have the training; but such patients for example they can be  known HIV patients who have been diagnosed with HIV long before they are diagnosed with MD-TB and in  such case of they were known to be RVI patients, such patients are those who have already initiated on the  drugs and when they come here we admit them here and make them to get drugs from their residence area  [KI means he refers to the facility where the patient was initiated on ART before coming to MDR-TB  treatment initiating center] or we link them to hospital here and facilitate the way that the patients can get  their drugs. Up to now there is no much problem in this regard. If they are new and diagnosed with HIV here that is if they are diagnosed both with MDR-TB and RVIand if we encounter such case, in nearby facility  there is Geda health center around here and we discuss with them and facilitate the condition whereby  patients can start their medication. Also in collaboration with the hospital we make sure that they get drugs.  Therefore just being here [at the MDR-TB center] patients can get their drugs. They get both the MDR-TB  and RVI drugs just here and there is no nay scenario whereby patients go to another facility to get drugs.  Mod: Do you mean that the RVI drugs for such patients are stored here?  KI: Correct. For each patient we bring two two month’s drugs at a time and refill every two month.  Mod: But do you feel that if both ART clinic and MDR-TB clinic is available here at this center, it facilitates  management of co-infected patients?  KI: Really, just now it is from another health facility that we are bringing drugs for our patients.If they can get ART  drugs at this center in same way they get MDR-TB drugs it would have been good if such arrangement is  made and in the future we will discuss with all concerned and try to facilitate the ways.  Mod: Do you have any additions that you would like for discussion and that I did not raise?  KI: No any. We have raised all that is needed.  Mod: Thank you. |
| 7. Caregiver (C.G. -7-MDR-TB physician | Mod: Thanks Doctor for your volunteering to be part of this study. Usually you initiate your MDR-TB patients on  treatment and stabilize them and then you link them to treatment follow up centers. What is your experience  regarding patients’ ability in getting continuum of care especially in getting daily DOT support at the treatment  follow up centers means that are providers there providing what is expected of them?  KI: Onmy behalf when I see the condition at the treatment follow up centers, our patients we initiate them at TIC  and when we send them to different TFCs we have been observing different things. Some TFCs or those places  where patients are linked after stabilized at our center, there are good conditions in some places they can properly provide the care when we observe, but in some places it can be as a result of shortage of persons it can also be  lack of deep understanding or knowledge about the subject matterI do not believe that TCs can properly manage  patients sent from TIC. It means that there are some places that have the knowledge and also some places that do  not have the knowledge. As the case of our surrounding or our work area there were many TFCs from those there  are those which are exemplary and there are also many that cannot fullyfollow and manage patients.  Mod; Do they give the daily DOT?  KI: There are some places that are more concerned and give that daily DOT. But on repeated visit there are some  places that fill out [the register] before providing the DOT, a one week drug given to patients home ahead and also  they deduct from what should be. From what I understand as a physician around this issue is that I understand the  presence of problem.  Mod: Up to now are there impacts on patients’ treatment outcomes from absence of continuous DOT support?  KI: In some places there are [impacts] .Specially at TFCs the way patients are handled. From this issue patients  there are incidences of conflict and compared with physicians at TIC, there are nurses at TFC majorly and they  disagree. As such there are patients that intermittently interrupt treatment. There are also patients who fully  socially agree with providers. Because of this TFCs are different from place to place and it is difficult for me to  generalize.  Mod: What I understand is that there are areas of strengths and gaps?  KI: Yes.  Mod: Apart from medication there are supports for patients like the nutrition and transport supports. As a physician  how do you understand that support?  KI: During my practice, these patients what makes them different from other patients like the HIV and diabetes is  that they have some supports. Even if I do not know the route as programmatic or other they have some support. This support includes transportation, this given to them as they repeatedly come for follow ups. Apart from this  there is nutrition or food support that they are given. What I understood at that time there is condition whereby  totally cash is given and patients take food that suits them only. But after a time it was changed to a system of total  food support. As a physician and as a physician treating patients, I do not support on my behalf. For example  those like the grain flour those not directly related with the MDR-TB and those given simply because it is food were  given. Therefore as an individual or as a physician I do not support much because patients should get variety food  and based on their interest; I have also seen that they want to select the type of food they should eat and they  [patients] have also reached to the point of conflicts. Therefore I do not think that it is proper.  Mod: As a physician which nutrients do you think that patients get, because what you tell me is food that are  source of more of carbohydrates? Which ones do you recommend for addition?  KI: Patients even for basic TB those treated for six months we need high protein dies. We get those high protein  diets from food items like the egg, milk and it can be from other foods like the beans. But the food items currently  given for MDR-TB patients more even if we see the food given for basic TB the foods that the programme is giving  for patients is not like egg, even though milk is available in the form of powder, it is not what patients prefer.  Therefore to say that it really builds their body and prevent their body from this disease even as the science states  it should be egg and milk or it is good if patients are given in the form of cash that I believe. Otherwise those like  the grain flour which are more of carbohydrates and those given in fractions I do not think that it is good.  Mod: What is your opinion on laboratory?  KI: On the laboratory, treatment of an MDR-TB it needs big laboratories those are sophisticated and big labs which  the patients cannot afford and which are not available in the laboratory. As my perception when I was treating  patients, there were many patients that were hurt because of lack of laboratory. For example thyroid function test,  electrolyte and such abnormalities and because others are directly related with the MDR-TB drugs that are done well in our hospital like the organ function tests are available. But from these like apart from the organ function test  like the thyroid function test and the electrolyte it would have been very good if it is available and done at the  hospital. In that way there were problems like the incidences of ‘sudden death that happened’. Thus both as an  individual and as a physician working there I was feeling the pain. But after this I think it may be improved.  Mod: I understand that the types of lab tests like the electrolyte tests that are important for patient management  and to prevent sudden death are not available in the hospital and also patients cannot afford the cost to get the  service from private providers and in that way there are cases hurt because of the absence of such lab tests?  KI: Yes  Mod: How do you describe the system’s support? When practicing as a physician do you feel supported by  immediate health management offices and your hospital management?  KI: During my service individually or based on own courage from being a physician and as a function of you  directly confronting patients and also usually it is your responsibility to take care of the patient as you repeatedly  visit and see the patient, the biggest responsibility is born on physicians and nurses working there. These groups  are those working day and night both to care for the patients and also to support the system. As per my service  setting the available support system is not encouraging. It may be because they do not go to and see the TIC, it  can be the hospital management or those working at zonal office there are forms of their involvement by especially  the hospital management‘s perception on MDR-TB is very low. Very much; so I don’t say that there is strong  support. It was damped only on the shoulders of one or two bodies, nurses and physician working there. I am  saying in such way because I encountered many times as I have been repeatedly treating patients and there also  were debates.  Mod: Currently it is reported that there is no comprehensive care facility in same compound to treat HIV and MDRTB  co-infected individuals. How do you observed that what do you recommend? KI: This is fact and very good point. From the many patients I had and patients I have been treating thirty percent  to forty percent had both HIV and MDR-TB. Especially HIV and MDR-TB whose mortality rate is very high and  patients chance to die is very high. When I see TB and HIV service is not located in one place. It is located in  different places. As a matter of chance because I have training both for TB and HIV I did not face much problem as  an individual. But because nurses working under my instruction and others do not know about HIV, we were facing  much problems. But not only here but on our TFC also it has been challenging us as they were not working the  collaboration. Therefore as to my view I say that it would be very good if the two services are available together in  one place. But the system around us because we do not have similar training and because we do not know much  about HIV, it has been challenging us to manage both diseases. But as an individual I believe that it is good if it is  located together and service provided together so that it brings change.  Mod: Finally is there anything that is not raised by me and that you want to raise?  KI: What I add is that treatment of MDR-TB is not something that starts at one point and stops at another point. I  believe that as other programmes, it should be incorporated into all the system like the diabetes and others and  many persons without exaggerating/fearing the problem and facilitating the situation for patents. Because MDR-TB  is seen as something unique and a disease not found in other places on earth and when I treat patients both  providers working there and the patients were perceived as unique persons. It should be seen as any other  disease and also if the system supports and integrates the service it would have been good.  Mod: You mean that all the disease, the provider and the patients are seen as unique persons?  KI: Yes, that is what I experienced. They also do not understand the work and because the service is also very  exhausting, it needs much support for those providing the treatment and those receiving the treatment. Therefore if  all from top to bottom collaborate I believe that they can prevent this disease.  Mod: specially you mean that the discrimination is all against the provider, the patient and the disease,.  KI: Exactly!  Mod: much thanks for the in-depth and clear discussion we had. Thanks, you  KI: Thank you for the chance you gave me. |
| 8) Caregiver (C.G. -8-MDR-TB Physician, Male | **Mod**: Thanks Doctor for volunteering to participate on this study. My first question is that, in the hospital you have been working at, patients after they are stabilized at your TIC, they are linked to catchment TFCs. Providers there [means at TFC] are expected to fulfill what is expected of them to enable patients continue getting the services they need there. What was your experience and observation in this regard?  **KI:** Yes patients are first are diagnosed at our hospital. After they are diagnosed, they are stabilized. So it is after they get better and are stabilized that they are linked to TFCs. After we send them the follow up there is good in some places and there are also facilities with gaps. On this we have been closely discussing on the problems and working with them. In majority of the cases it is good. But there is gap in some health centers. There are problems whereby the provider fears approaching the patients and problems of giving incorrect drug dosage. We have discussed on those problems and there are problems that are solved but there are areas still where we have fears.  **Mod:** You rose that in some areas the providers fear approaching the patient and problems of incorrect drug provision. Rarely there practical problems resulted as the consequences of such practices? What you recommend to address such challenges?  **KI:** Yes, for example because of fear of the patient there are incidents whereby the patient discontinued drugs. A patient discontinued for three weeks and for the time what we did was that we returned the patient to our hospital [TIC] and then it was after we went there and gave the health care providers all the available information that the patient was re-linked to the health center. On this issue it is advisable to equip the health care provider with the necessary information after the patient is linked to the TFC to prevent patient’s disappearing from treatment when providers fear approaching them.  **Mod;** You mean that the cause of such problems is lack of awareness?  **KI:** Yes it is problem of awareness.  **Mod:** Thanks! The next discussion point is that there is scheme called patient treatment enablers. There are services there done through like covering cost of transport or nutrition support. How do you describe the strengths and gaps you observed there?  **KI:** To mention the strengths, there is budget. Budget is allocated. There is budget allocated by the health bureau for nutrition support and there is budget. The gap it has is that gaps are available on hospital in properly utilizing these budgets. They spend the budget for different reasons. This is the big problem I faced in my hospital. The patients do not get the whole financial benefit they deserve from the budget. It is good if tracking or monitoring system exists on that I mean that the problem is not only from the hospital, even the health bureau when we tell the problems it does not follow and solve the problems. The health bureau itself, Oromia health bureau did not solve the problem knowing existence of such problems. Down there is problems, there is problem that patients do not get the benefit fully so that it is better if it is acted up on the problem in collaboration.  **Mod:** I understand that resources allocated are not utilized properly for the benefit and support need of patients.  **KI:** yes, yes! It is not enough. They do not access as it is allocated for them.  **Mod:** During patient treatment one issue is use of laboratory. You need laboratory to monitor patient response to treatment. In that regard what conditions are prevailing doctor?  **Mod:** There is big gap on the laboratory as well. Samples sent for culture do not reach to labs on time. Some samples disappear in between. Normally we send every month and track it. Once the patient starts treatment and we send sample for culture, the result does not turn back on time and sometimes four months and sometimes six months that it stay. This condition puts us at difficulty to know whether the patient is cured or improving with the treatment. this is the existing gap. Secondly |
| 9) Caregiver (C.G. -9-MDR-TB Nurse from TFC | Mod: Thank you for consenting to participate on this interview. You are providing care for patients with MDR-TB. These patients take drugs for about two years; and they also need to attend to the daily treatment under direct observation by treatment supporter like you. What are the practical challenges you faced so far regarding assisting patients with MDR-TB to adhere to this lengthy treatment and how do you mitigate the challenges to enable patients continue their treatment?  KI: In our context, the problem I can mention as critical is on patients taking drugs daily under DOTS, our patients come from remote rural areas; it is difficult to make sure that their attendance to treatment is fully adequate. If I speak for the sake of the truth based on the practical recommended, it is a barrier for us, it is really a barrier. For the daily injections, there are challenges. So far two patients completed that treatment at our disposal and it was through many challenges that we succeeded in enabling patients complete their treatment. Again the current patient with me who takes the drugs is also from same MDR-family (means, household contacts of those who completed treatment). Same challenge we faced with those completed treatment is that we face with this new patient, however as mentioned earlier, in the area of treatment the patient will not get adequate support in the absence of health trained worker. Absence of training for caregivers is considered as problem; also providing service with only one provider is a problem. It is considered as an individual business, as the personal business of the caregivers who is assigned to the service point, and usually it is not seen as the responsibility of the whole health worker of the institution, it is seen as my personal business. Yet, there has been a big problem at start, but now I perceive a small improvement at least in considering the presence of the problem. The focal person of the district health office there is some attention and our supervisor also give some attention nowadays.  Mod: It is good that there is some improvement in the attention provided by immediate supervisors as you mentioned. Yet, I perceive that for patients who come from remote rural areas, it will continue to be a problem to attend to their daily treatment at the heal center level. How do you suggest that this problem can be addressed in practical terms?  KI: In terms of attention, the practical solution I suggest is that I recommend that patients who come from remote areas need to have the opportunity to rent house in the hometown of the health center.  Mod: Now because there is no accommodation facilities, do you mean that they come from their home which is remote areas on daily basis.  KI: Yes, they walk a two hours journey on daily basis to take their daily injections .  Mod: Have their been incidences whereby patients has discontinued treatment so far because of the distance from health center?  KI: No, no so far, but sometimes there are incidences whereby rivers overflow and they could not cross the reiver during rainy season, and they miss a two- or three-days drugs,  Mod: Do you mean that this problem is encountered by your facility during rainy season?  KI: Yes, many times we try to do all we can and in most cases we our selves could not do anything to help the patient and the drugs are missed. We tell patients the danger associated with missing drugs and even the drugs should be taken at similar time on daily basis.  Mod: During their attendance to treatment at your facility patients with MDR-TB may encounter drug-related adverse reactions, have you encountered patients with adverse drug reactions so far, and if so how did you manage them; once a monthly basis they go to hospital but the stay at TFC for the rest of the days, Therefore how do you manage side effects if they occur?  KI: There has not been severe problem, but sometimes there is problem of gastritis and diarrhea and there is time when I prescribe them ORS and also, I provide them with PLampy Nut when we have some. There is also vitamin B complex and when their appetite is poor I provide them with vitamin B complex. And it means that sometimes, there is also times when we discuss with OPD clinicians and provide a care that the patient may need for side effects  MOD: Regarding laboratory services, for patient diagnosis and laboratory follow up services for patients those on treatment and whose samples are sent for follow ups, is there any challenge that you faced and how do you deal with the problem?  KI: At this time our laboratory is fully functional!  Mod: How is the management of patients with MDR-TB is perceived? Is it perceived as problem of an MDR-TB caregiver, a problem of health center manager or district health office or issue of all concerned? Do health center management and the district health office support you on the issue, do you feel supported by them; are you recognized for the care you provided for such patients?  KI: It does not mean that I am dedicated for the MDR-TB service only, sometimes when I am on duty overnight and day off other staff provide injections for patients. When I am day of I request them to support the patients and as staff there is no much problem there is sense of supporting one another.  MOD: So far have you encountered a patient with MDR-TB who is also infected with HIV?  KI: No so far I have only one TB patient who even was on HIV care for many years and then diagnosed with TB.  MOD; You mean that you do not have MDR-TB patients co-infected with HIV?  KI: Yes, I do not have so far.  Mod: You know that there is nutrition support for patients with MDR-TB and also support on const of transport through hospitals, do you perceive that that nutrition support is enough? From your daily encounter, what do you hear and perceive from patients you treat?  KI: I only know that patients are supported through hospitals. I never investigated the issue but some patients talk that when they were at hospitals, they were given food and accommodation services, and they ask me whay the government continue the accommodation support so that we can rent houses and attend treatment at the health center, they ask me this question, that is because of their distance from the health center.  Mod: Do you have any points that you perceive that I did not present for discussion?  KI: I do not have any additional point but as a provider I will continue supporting patients without any conservation as long us my God gives me health and the strength, But the question I have is that what is the risk allowance that we get, we are treating the patients and even TB patients who we were treating has developed MDR-TB and also we are treating their MDR-TB, how is this problem is considered by those responsible?  Mod: Do you mean that as you are engaged in risky clinical services, a risk allowance incentive should be given for such caregivers?  KI: Yes. |
| 10) Caregiver (C.G. -10- MDR-TB Nurse from TFC | Mod: Thank you my sister for volunteering to participate on this interview with patients with cargivers for MDR-TB. I hope that you have provided treatment services of patients with MDr-TB and also doing so at this time. Provision of daily treatment under observation for such patients and for the patioed of 2 years is may be a huge task. How do you explain your experience on such issues?  KI: Dealing with patients with MDR-TB is a challenging task, I mean that when we compare it with other diseases. The drugs they take on daily basis is up to eight months, because they take drugs and the also have injections I mean. After that even if we expect them to come to TFCs to take drugs on daily basis, they will not volunteer for that, it means for two to three days we give them the drugs so that they take the drugs at their home.  MoD: You mean that they will not come to the health center on daily basis for the whole period of the two years>  KI: Yes, they will not come daily for the two years period. There is the so called TTS for TB, it means that I make use of them to assist patients.  Mod: The TTS’s are usually from family members of the patients with MDR-TB and the number of anti-microbials taken by the patient on daily basis is huge both in terms of the kind of the drugs taken and the number of tablets they take which may be around fifteen to sixteen tablets per day, can they differentiate the drugs? How did you understood that?  KI: Differentiation of the drugs, I will teach them, when the family the TTS I use to use them to assist patients to provide the drugs, There is a form that the TTS fills out, there is a card and I make the TTS sign on the card after I teach them, the patient also precisely knows the drugs he takes, and also knows the time the drugs are taken and there is no such much problem with that.  Mod: When you provide daily tablets, how many tablets an individual takes in one day?  KI: One person takes may be fifteen it may also be sixteen, it depends on the patient.  Mod: Over what time period do you provide the whole tablets for a patient?  KI: At least it means that we will finish all tablets in about thirty minutes, it means that he takes the drugs and take a rest in between. As it is difficult to take at once, he takes the drugs by taking rests, when he also takes the drugs at home we tell him to take rest in between while swallowing the drugs, we tell them.  Mod: I understood that you provide drugs on daily basis during first eight months and then provide a two to three days drugs so that the patient takes the drugs at home. KI: Yes! Mod: Mod: You also use TTS, and in this regard what were the challenges that you encountered from patients or from providing the services and how do you overcome the challenges?  KI: There are challenges, the drugs are many, the patient is bored with taking the drugs; moreover the side effect of cycloserine it changes their behavior different from behavior of a normal person. Now, a good person changes his behaviour and develops a new behaviour. These patients come and if I am not at the health center, they even do not want to be treated by other caregiver. They complain pains and they come up with different complaints.  MOD: Do you maen that, once they get acquainted with you, they do not want to deal with other care providers,  KI: Yes, they do not want to talk with others, they may quarrel with other caregivers on not wearing face mask like that. Enduring all these challenges, we assist the patient and help them to complete their treatment.  Mod: do you mean that patient on the side of the caregiver is important?  KI: It is very important. In addition to the drugs, they also wear masks and they feel when they become identified from other patients and they feel; wearing the mask and waiting for the service, they feel anxious. Therefore it needs to keep the patients close as much as possible.  Mod: What drug-related side effects did you encounter so far and how did you manage them?  KI: I did not encounter severe side effects; they are minor side effects and those I treat there it means; I did not come across severe side effects.  Mod: At your health center, how is the services given for patients with MDR-TB is perceived, whose responsibility is it as perceived by co-workers and your supervisors? Do you feel thay you are supported by the nmanagement of your health center and by the district health office?  KI: They support me but, it is me who is personally responsible for the services, when drugs are stockout it is me who contact the hospital and make the drugs to reach for me, they support me but it is not this much. It is me who is personally responsible for taking care of the patients.  Mod: is there anything that you would like to raise but I did not raise during this discussion?  KI: In majority of the cases, ours do better and I cannot say that the leave the service to me. It is me who usually make things happen, otherwise there is no such much problem and there is no a problem that I encountered and that they left for me.  Mod: you are providing the care that patients need at your disposal, is there anything that you suggest that if it exists the services for patients would be improved?  KI: to some extent, when they come from hospital a transport cost is given for the patients and there are other supports that patients get, but they are not satisfied with that as such. They say it is not enough, what can this work for me they say. Indeed if that support is not available I do not think that they will go to the hospital monthly; if they are not paid transport cost, I perceive that they will not come back, thay need to be taken into consideration.  Mod: Is there anything that you may add?  KI: No, nothing left, but this needs the commitment all people. From top to bottom on MDR-TB to reduce it everyone need to commit its share responsibility.  Mod; Thank you for your time and your valuable ideas that you shared with me.  KI; thank you. |
| 11) Caregiver (C.G. -11-MDR-TB Nurse from TFC | Mod: Thank you for volunteering to be part of this discussion. Let us discuss that you have patients treated for MDR-TB, and the patients need treatment for about two years, there are many encounters that you may have experienced like the issue of daily patient treatment under observation, and in relation to this what are the practical situation that you have experienced in the context of your environment?  KI: Now what is seen practically is, the patients we have we received them from hospital, because there were problems that exists for example the TB clinic and general injection room is same in our case, general injection service I mean. A scenario that doesn’t exist anywhere I mean, and to separate it I have told responsible body and we talked over many things to correct it and the bureau also knows the issue, other higher bodies also know the issue as in the case of our facility. For this reason, it will be corrected, a room will be built that we were told, otherwise there is no any correction taken so far, the two services are not separated so far. Because of this ours patients, it is the hospital who took over and give the treatment. Just today we are here and it is to reengage us in the provision of the services. MDR-TB, although the patients are ours, we are not providing the services, we could not provide the treatment.  Mod: If MDR-TB patients come to your facility, you mean that they get service in same room where other patients are given injection services?  KI: Yes! This problem, very much has challenged us even to start provision of the service.  Mod: When you strive to provide care for patients with MDR-TB, how do others like the health office and health center management perceive the issue including to solve the problems you stated, I mean?  KI: Now, because they think that the problem is of my concern only. That is why they didn’t solve the problem we have as I perceive for myself. I talk always, but they have problems in solving the problem; our managers I mean, I talk always, those who work with e also raise the issue but there is huge problem in deciding on the issue, they consider that the task as my only task. Otherwise, they about MDR-TB and about TB they have enough understanding. They see us only because they do not want to give attention to the issue.  Mod: You mean that your managers do not give the needed ttention to make decisions and solve the problems you have?  KI: Yes.  Mod: The other issue, what challenges did you face so far regarding TB and MDR-TB laboratory?  KI: Regarding laboratory, problems we have those who can do laboratory work but we do not have the trainings, they are not trained. It is just now that they have planned to train them starting from the upcoming Monday. They are planning to send our laboratory. Otherwise no such much problem that I encountered apart from the trainings  MOD: Okay, heve you enever seen an MDR-TB patient who also had HIV so far?  KI: Not MDR-TB. Those who had TB and also HIV That we had so far.  Mod: For such patiens, if they get TB treatment from TB clinic, from where they get the treatment they need for HIV  KI: It is just from our facility. Because our facility is ART centered, they get from ART clinic. Others who did not disclose their status and who are referred from other places, they take the from their referral facilities, we encourage them to start taking from our facility we give them education, some of them have the will but others do not want to take from this facility and they continue where they start the (HIV) treatment  Mod: Otherwise, it means that you do not have training on ART  KI: Yes, I do not have training on ART  MoD: when your patients take treatment from TB from your room, they need to visit other facilities for the ART.  KI: Yes,  Mod: If you take the training on ART do you think that you can provide the ART service at TB clinic? Do you think that it is good fro TB patients?  KI: As the number of TB patients I am supporting are many, I think that that is good for the patients.  Mod: Thank you fro your ideas, and is there anything that you want to add to what already said?  KI: The things that I may add no, but if you can do that let you assist us in solving the issue in our facility. If it can be separated from each other, and those who caught can be supported, I am happy if you may support us with that.  Mod: Thank you! What you raised is infact an issue that needs consideration.. Thank you for your time and your comments. |
