# Supplementary material for: Patients’ perceived quality of care and their satisfaction with care given for MDR-TB at referral hospitals in Ethiopia
Source: PLoS One. 2023 Feb 2;18(2):e0270439. doi: 10.1371/journal.pone.0270439 (PMC9894439; doi:10.1371/journal.pone.0270439)
Supplement: Attachment — Submitted filename: Comments.docx [file pone.0270439.s002.docx]

**Dear Editor of PLOS ONE Journal,**

Many thanks for your invitation to review the Manuscript. Due to the scientific value and originality of the subject, it is greatly suggested that the manuscript is accepted for publication in the Journal with minor revisions (Please refer to the comments below).

**In general:**

- Ethical approval number for collected samples from volunteers should be provided.
- The quality of English writing is good, but some grammatical and structure errors should be revised.
- It is not recommended to write any abbreviation in manuscript title
- Any abbreviation should be written in full name for its first description, thereafter, the abbreviations only are mentioned.
- Common antimicrobials used for MDR-TB treatment of patients should be provided
