# Supplementary material for: Patients’ perceived quality of care and their satisfaction with care given for MDR-TB at referral hospitals in Ethiopia
Source: PLoS One. 2023 Feb 2;18(2):e0270439. doi: 10.1371/journal.pone.0270439 (PMC9894439; doi:10.1371/journal.pone.0270439)
Supplement: Attachment — Submitted filename: PONE_16727.docx [file pone.0270439.s003.docx]

**Patients’ perceived quality of care and their satisfaction with care given for MDR-TB at referral hospitals in Ethiopia**

**General comments**

The review has been done in two steps:

1. The manuscript was reviewed exhaustively and the comments/queries were broadly given under each subheading.
2. Secondly, COREQ – Consolidated criteria for reporting qualitative research was employed as a standard checklist.

**Introduction**

There is a duplication of definitions of perceived quality and patient satisfaction in terms of “patient’s expectations and their perceptions on the quality of services”. Hence, this could be clarified.

Secondly, as this is a study from Ethiopia it would be helpful if the author could provide details on the current burden of MDR TB in Ethiopia which will help the reader to understand the context.

*Thirdly, the introduction could be divided into the following paragraphs as:*

1. MDR-TB in Ethiopia and points related to the treatment in terms of time, cost and tolerance including an emphasis on the functionality attributes and the operation of the programme in Ethiopia.
2. Related to patient’s perceived quality of care and their satisfaction.
3. Literature related to the above with special emphasis to MDR-TB

**Materials and methods:**

**The heading of materials and methods** is missing under which setting, study population and participant selection should be done.

As it is qualitative research there were few critical points which are missing like:

1. Inductive or deductive approach was employed
2. Type of qualitative approach, whether ethnographic/narrative/grounded theory or phenomenological?
3. The subheading should ideally be “study setting” and study period.
4. Was there any exclusion criteria? As the number mentioned 23 patients were identified out of 136
5. Was any reference/approval number of protocol or letter of permission from the ethics committee being obtained?
6. Who were the interviewers (mentioned as initials)
7. Was the interview audio recorded or field notes were taken?
8. What was the language in which interviews were taken and were it translated ?
9. How much was the total duration of the interview?

**Results**

1. Abbreviations could be given at the end of the manuscript or somewhere while being used for the first time within the text for letters like P – participants.
2. As it is a thematic analysis, the addition of a thematic framework might be considered to get a wholesome idea.
3. Certain sentences in the results section could be added to the introduction like the regional health bureau bearing the references. Also, the functionality of the MDR TB programme has already been discussed in the introduction and hence a crisp summary would suffice in the results section.
4. The results section can begin with what were the 7 themes that are to be discussed.
5. A brief mention of sub-themes under each theme can be highlighted because the data analysis section had mentioned 29 sub-themes, but they were not evident in the results.
6. There were repetitions pertaining to the nutritional and socio-economic status in the health systems theme as well as in the socio-economic theme. The concern from the stakeholders like 3 physicians and 8 staff nurses regarding the above can be captured as an excerpt in the health systems part.
7. There has been over-emphasis regarding acquiring the TB disease among the household contacts. However, a brief write-up about this concern and a suitable verbatim in any of the results section could be considered.
8. Verbatims for the following theme on Respiratory MDR Tb infection control practices are missing.
9. As the study is on patients’ perception on quality of care and satisfaction, the inclusion of suitable verbatim might be added.
10. Also, there were few verbatims that could be added in favour of satisfactory services and good quality of care otherwise the manuscript will give the reader “*reporting bias”*.

**Discussion:** Could be better organised along with the flow of themes.

**Other comments as per the COREQ checklist has been provided below.**

| **Section** | **Personal Characteristics** | **Whether it is reported** | **Reviewer comments** |
| --- | --- | --- | --- |
| **Domain 1** |  |  |  |
| **Research team and reflexivity** | |  |  |
| **1** | Interviewer / Facilitator | Which author/s conducted the interview or focus group? | Given in general under author’s contributions in pg.12 but not mentioned in data collection and results.  The initials could be noted in the data collection part for e.g., MK |
| **2** | Credentials | What were the researcher’s credentials? E.g., PhD, MD | Mentioned for all except Mavhandu Mudzusi |
| **3** | Occupation | What was their occupation at the time of the study? | Mentioned |
| **4** | Gender | Was the researcher male or female? | Not given |
| **5** | Experience & training | What experience or training did the researcher have? | Not been mentioned |
| **Relationship with participants** | |  |  |
| 6 | Relationship established | Was a relationship established prior to study commencement? | Not mentioned |
| 7 | Participant knowledge of the interviewer | What did the participants know about the researcher? e.g., personal goals, reasons for doing the research | Only objectives of the study have been provided as given on pg.3 |
| **8** | Interviewer characteristics | What characteristics were reported about the interviewer/facilitator? e.g., Bias, assumptions, reasons and interests in the research topic | Not reported. |
| **Domain 2: Study design** | |  |  |
| **Theoretical framework** | |  |  |
| **9** | Methodological orientation and Theory | What methodological orientation was stated to underpin the study? e.g., grounded theory, discourse analysis, ethnography, phenomenology, content analysis | Not mentioned |
| **Participant selection** | |  |  |
| **10** | Sampling | How were participants selected? e.g., purposive, convenience, consecutive, snowball | Mentioned under study population and participant selection as shown in pg.3. |
| 11 | Method of approach | How were participants approached? e.g., face-to-face, telephone, mail, email | Not mentioned |
| 12 | Sample size | How many participants were in the study? | Mentioned under the study population in pg.2. |
| 13 | Non-participation | How many people refused to participate or dropped out? Reasons? | Not mentioned. |
| **Setting** | |  |  |
| **14** | The setting of data collection | Where was the data collected? e.g., home, clinic, workplace | Mentioned as referral hospitals under setting in pg.2. |
| 15 | Presence of non-participants | Was anyone else present besides the participants and researchers? | None |
| 16 | Description of sample | What are the important characteristics of the sample? e.g., demographic data, date | Not detailed enough as given in first paragraph below results section. |
| **Data collection** | |  |  |
| 17 | Interview guide | Were questions, prompts, and guides provided by the authors? Was it pilot tested? | Only mentioned as semi-structured interview on pg.3.  No mention has been made of pilot testing |
| 18 | Repeat interviews | Was repeat interviews carried out? If yes, how many? | Nil |
| 19 | Audio/visual recording | Did the research use audio or visual recording to collect the data? | Nil |
| 20 | Field notes | Were field notes made during and/or after the interview or focus group? | Not mentioned |
| 21 | Duration | What was the duration of the interviews or focus group? | Not mentioned |
| 22 | Data saturation | Was data saturation discussed? | Not mentioned |
| 23 | Transcripts returned | Were transcripts returned to participants for comment and/or correction | Not mentioned |
| **Domain 3 – Analysis and Findings** | |  |  |
| **Data analysis** | |  |  |
| 24 | Number of data coders | How many data coders coded the data? | Not mentioned |
| 25 | Description of the coding tree | Did the authors provide a description of the coding tree? | Not mentioned |
| 26 | Derivation of themes | Were themes identified in advance or derived from the data? | Derived from the data as given under data analysis section in pg.3. |
| 27 | Software | What software, if applicable, was used to manage the data? | Mentioned to be done manually under data analysis in pg.3. |
| 28 | Participant checking | Did participants provide feedback on the findings? | Lack of feedback was addressed in pg.9 as one of the interesting finding. |
| **Reporting** | |  |  |
| 29 | Quotations presented | Were participant quotations presented to illustrate the themes/findings? Was each quotation identified? e.g., participant number | Yes, as mentioned in the results section in italics from pg.4. |
| 30 | Data and findings consistent | Was there consistency between the data presented and the findings? | Yes.  But the study should also capture findings that were in favour of satisfactory practices and good quality to avoid reporting bias. |
| 31 | Clarity of major themes | Were major themes clearly presented in the findings? | Yes.  Has been mentioned in the results section from pg. 4. |
| 32 | Clarity of minor themes | Is there a description of diverse cases or a discussion of minor themes? | It has been submerged within the major themes.  Hence, highlighting the minor themes would provide clear picture to the reader. |
