# Supplementary material for: Patients’ perceived quality of care and their satisfaction with care given for MDR-TB at referral hospitals in Ethiopia
Source: PLoS One. 2023 Feb 2;18(2):e0270439. doi: 10.1371/journal.pone.0270439 (PMC9894439; doi:10.1371/journal.pone.0270439)
Supplement: Attachment — Submitted filename: Response to Reviewers.doc.docx [file pone.0270439.s004.docx]

September 19, 2022

Mengistu Kenea Wakjira

Mobile: 251-91-1-30-25-68

P.O.Box: 28,398/1000

E-mail: mengistukenea@gmail.com

Addis Ababa, Ethiopia

The PLOS ONE academic editors/reviewers’ team

Dear Madam/Sr:

Warm Greetings!

This letter is in reference to reviewers’ request to include a rebuttal letter that responds to point raised by the academic editors. First, I want to owe my heartfelt thanks and appreciations for the reviewers for their dedication to assist me to improve the quality of the manuscript being processed for publication. My responses to the points raised by the editors are indicated in the table that follows.

Best regards

Mengistu Kenea Wakjira

# **Table of responses to points raised by academic editors/reviewers.**

| Reviewers’ comments | Explanation from the authors |
| --- | --- |
| **Introduction section** |  |
| Write abbreviations in full the first time they are mentioned in the body of the manuscript | Whenever they are used, abbreviations are written in full the first time they are mentioned in the body of the manuscript. |
| Provide the burden of MDR-TB in Ethiopia | This is addressed as per this paragraph: “According to Biadglegne et al 2014, Ethiopia is among the 30 countries in the world described by the World Health Organization as high burden for TB and MDR-TB. Thus, the government rolls out community-based programmatic management of drug-resistant TB across all its provinces.” |
| Ethical approval number for collected samples from volunteers should be provided. | Participant information sheet and consent form used during participant selection is attached |
| Revised grammatical and structural errors | Revisions are made to correct any grammatical and structural errors |
| Duplication of definitions of perceived quality and patient satisfaction in terms of “patient’s expectations and their perceptions on the quality of services”. | This is revised and clarified throughout the body of the manuscript |
| **Materials and methods section** |  |
| Letter of permission from the ethics committee | Ethical approval from institutional board and letter of permission from relevant health department’s research directorate is obtained and it is uploaded |
| The type of qualitative approach used to conduct the study | An inductive phenomenological approach was used in this study to understand the contextual lived experience of patients with MDR-TB and their caregivers |
| Subheadings should identify “study setting” and “study period” | This comment is addressed in the materials and methods section of the manuscript |
| How were participants approached for interview (face-to-face; telephone, mail.) | Interviews were made by the principal investigator (MK) through **face-to-face interviews** with patients with MDR-TB. This is mentioned under data collection section of the manuscript. |
| Presence of a non-participant during individual interviews | A trained note-taker was taking notes on the feelings and experiences of the study participants. This is mentioned in the data collection section of the manuscript. |
| Selection criteria | Information adults aged 18 years and above who can consent for the interview were eligibility criteria. Information was not obtained from those aged under 18 years! |
| Who were the interviewer | The principal investigator (MK Wakjira) conducted the in-depth interview with patients and their caregivers. The principal |
| Was the interview audio taped and notes taken | Interview data were audio recorded and transcribed verbatim. Notes were taken during interviews where data were captured on the feelings and experiences of the study participants. Data from multiple sources were used to make sure that emerging themes are established based on converging different sources of data or different perspectives of the study participants. |
| Description of the study sample | A total of eighteen adult patients with MDR-TB; 9 (50%) female and 9 (50%) male patients were included in the study. Three of the 18 (17%) participants openly disclosed that they were MDR-TB and HIV co-infected. Moreover, eleven caregivers for MDR-TB (3 physicians and 8 nurses) participated in the in-depth interviews. |
| Language used to for the interview | The patients mother tongue language (Afan Oromo and Amharic) was used for the interview. As an Ethiopian national, the interviewer (MK Wakjira) is proficient in both languages and language. |
| Duration of each interview | Each individual interview was conducted for about 30-40 minutes (this is indicated in patients information sheet and consent form uploaded with the manuscript. |
| Was data saturation discussed | Yes! As discussed under “study population and participant selection” section, the number of participants of the interviews was determined by category saturation, the point at which the interviews did not reveal new data relevant to the objectives of the study |
| Are transcripts returned to participants | Yes. Member checking was employed with each participant in which summarized interview data were presented to each interviewee and corrections sought to understand if what the researcher captured matches the intentions and opinions of the participant. Transcripts were also given to some literate participants, and they were asked if what the researcher captured matches their opinions. Moreover, two debriefers who had experience in social research were located and their frequent feedbacks were used to enhance accuracy of the construct under scrutiny. |
| Data Availability | Authors declare that data underlying the findings of this study is available without restriction. The whole verbatim transcript of the qualitative data is uploaded |
| **Results section** |  |
| Include common antimicrobials used to treat MDR-TB | Included under the section “Adverse drug reactions and its management” |
| Abbreviations could be given at the end of the manuscript or somewhere while being used for the first time within the text for letters like P-participants | This is addressed starting from the “abstract page” of the manuscript.  For letters like P-participants and C.G-caregivers a footnote I used to illustrate them on the same page where the letters are used as abbreviations. |
| Provide common antimicrobials used in the treatment of MDR-TB | List of common antimicrobials used in the treatment of MDR-TB is provided under the section dealing with “adverse drug reactions from drugs used in he treatment of MDR-TB” |
| Begin the results section with major and sub-themes | This comment is addresses in the result section |
| There were repetitions pertaining to the nutritional and socio-economic status in the health system theme as wells in the socio-economic theme. Concerns from participants can be captured as an excerpt. | The repetitions are revised and corrected. The concerns and views of patients with MDR-TB is clearly raised and it is also indicated that there is no feedback from the system on the concerns and views of patients with MDR-TB. |
| Over-emphasis of acquiring MDR-TB among household contacts. Add a brief write up on this | A brief writes up including excerpt is provided in this section |
| Verbatim for respiratory MDR-TB infection control is missing | An excerpt illustrating the risk of and situation of respiratory MDR-TB infecting is included |
| The study is on patients’ perception of quality of care and satisfaction, inclusion of suitable verbatim might be added | An excerpt is added to illustrate patients’ perception of the quality of care they received at hospitals and at the community level y treatment follow up centers. Excerpt is also added to make similar illustrations on the non-clinical (socio-economic) services and support that patient got from the programme of MDR-TB |
| Reports on satisfaction to avoid reporting bias | This is clearly reported under the title of “Patients’ perceived quality of care and their satisfaction with the care given for MDR-TB”- At hospital level, the communication between patients and their caregivers was revealed to be optimum. Thus, the majority of patients were satisfied with the smooth communication and empathetic clinical care they received at hospitals…. |

| **Discussion** |  |
| --- | --- |
| Could be better organized along with the flow of the themes | This comment is addressed in the discussion session. The discussions are reorganized along with the flow of the themes in the study. |
